# Estimation of plant leaf water content based on spectroscopy

**DOI:** 10.3389/fpls.2025.1609650

**Published:** 2025-06-02

**Authors:** Jiangtao Ji, Xinyi Lu, Hao Ma, Xin Jin, Shijie Jiang, Hongwei Cui, Xiaoxuan Lu, Yaqing Yang

**Affiliations:** College of Agricultural Equipment Engineering, Henan University of Science and Technology, Luoyang, Henan, China

**Keywords:** plants, leaf water content, hyperspectral technology, vegetation index, generalization ability, cross-species validation

## Abstract

**Introduction:**

Leaf water content is a key physiological indicator of plant growth and health status. Constructing leaf water content estimation models based on spectroscopy is an effective method for monitoring plant physiological conditions.

**Methods:**

To improve the accuracy of leaf water content estimation and develop models applicable to different plants, this study collected 1,680 groups of hyperspectral and water content data from peach tree leaves. Estimation models were established using two methods: “constructing vegetation indices” and “selecting characteristic wavelengths.” The accuracy and number of wavelengths used in each model were systematically evaluated. The optimal model was used to predict the water content of each pixel in the hyperspectral images, achieving visualization of leaf water distribution. Additionally, 244 groups of hyperspectral and water content data from apple tree and lettuce leaves were collected to validate the generalization ability of the optimal model.

**Results:**

Results showed that the optimal models established using the two methods were the linear regression model based on the vegetation index NISDI (3 wavelengths, R_P_^2^ = 0.9636, RMSEP=0.0356), and the CARS-RF model (12 wavelengths, R_P_^2^ = 0.9861, RMSEP=0.0219). Although the accuracy of the two models was similar, the latter used four times more wavelengths than the former, so the former was chosen as the optimal model. Using the optimal model to estimate the water content of apple tree leaves, the R_P_^2^ and RMSEP were 0.9504 and 0.1226, respectively. For lettuce containing only leaf tissue, the R_P_^2^ and RMSEP were 0.8211 and 0.1771, respectively.

**Discussion:**

These results indicate that the model has some generalization ability and can accurately estimate the water content of leaves of woody plants in the same family, with some performance degradation across different growth forms. The study results achieved accurate estimation of leaf water content for three types of plants and also provided a reference for establishing plant leaf water content estimation models with generalization ability.

## 1 Introduction

Leaves are the principal sites for photosynthesis, transpiration, and other physiological processes in plants, and their condition is directly linked to the overall health and productivity of the plant. Research indicates that the water content in plant leaves typically ranges from 40% to 80%, with varying optimal leaf water content for different plant species and even within the same species at different growth stages. Excessive or insufficient leaf water content can impact photosynthesis, respiration, and overall plant growth (Qu et al., 2018). Consequently, the scientific estimation of plant leaf water content is of significant importance. Leaf water content is influenced by a multitude of factors, including soil moisture, climate, and light, which makes rapid and accurate measurement challenging (Wang et al., 2022). Conventional methods for measuring plant leaf water content, including the oven-dry weight method (Peng et al., 2020), Karl Fischer titration (Horablaga et al., 2023), and the capacitance method (Lu et al., 2022), provide accurate results but are laborious and time-consuming, limiting their widespread adoption. Therefore, how to rapidly and accurately detect the water content of plant leaves, monitor the physiological status of plants in real-time, and provide timely warnings of water stress remains a current hot issue.

Hyperspectral technology has witnessed rapid development in recent years, leveraging its advantages of efficiency, rapidness, non-destructiveness, and environmental friendliness (Yang et al., 2023b), and has become a key research direction in the field of plant leaf water content detection. Current detection methods mainly include physical model methods and empirical model methods. Physical models based on radiative transfer mechanisms, such as PROSPECT and PROSAIL, have clear physical meanings and higher stability. However, they require many precise leaf structural parameters as inputs (Wang and Li, 2012), and the model construction and parameter optimization processes are complex. In contrast, empirical model methods typically establish regression models based on the relationship between spectral data and leaf water content. They do not require an understanding of the leaf’s internal structure and biochemical composition. The model construction process is relatively simple and computationally efficient, making them more suitable for real-time and rapid detection.

Scholars often use characteristic wavelength bands to construct empirical models for estimating plant leaf water content. Dai et al. (2022) utilized hyperspectral technology for the rapid and non-destructive detection of water content in citrus leaves, employing pseudo-color processing to visualize water content. A comparison of various feature wavelength selection and modeling approaches was undertaken, and it was determined that a CNN model built with 29 feature wavelengths selected by CARS yielded the best results, with R² and RMSE values of 0.9679 and 0.0163, respectively (Liu et al., 2024). introduced an interval variable iterative spatial shrinkage method (IVISSA) combined with interval partial least squares (iPLS) for identifying feature wavelengths, selecting 30 feature wavelengths to establish a least squares support vector regression (LSSVR) model for the rapid and non-destructive detection of water content in rapeseed leaves, achieving prediction set results of R_P_^2^ = 0.9555 and RMSEP=0.0065. Dong et al. (2022) applied hyperspectral technology to detect water content in withered black tea leaves and created moisture distribution maps under varying degrees of withering. The optimal model, SNV-Si-CARS-ELM, achieved R² and RMSE values of 0.9940 and 0.0074, respectively, offering a theoretical foundation for the rapid and non-destructive detection of water content in withered black tea leaves. Lv et al. (2024) gathered reflectance data from catalpa leaflets via hyperspectral technology and developed several estimation models for catalpa leaf water content using diverse variable selection and model construction techniques. The MC-UVE-PLS model was found to be the most effective, with an R² of 0.7903 and an RMSE of 1.7352. Zhang et al. (2023) collected spectral data within the 900-1700nm range from rapeseed leaves using near-infrared hyperspectral imaging and proposed employing convolutional neural networks (CNN) and long short-term memory (LSTM) to predict water content in rapeseed leaves, with test set R_P_² and RMSEP values of 0.8140 and 0.0050, respectively.

Some scholars have also established empirical models by constructing vegetation indices to estimate leaf water content. To predict the water content in citrus leaves, Dou et al. (2024) collected hyperspectral data and discovered that utilizing Continuous Wavelet Transform (CWT) decomposition with SPA for feature wavelength selection, combined with the normalized difference vegetation index (NDVI) $\left(R_{800}-R_{680})/(R_{800}+R_{680}\right)$ for model construction, resulted in the best estimation performance, with R² at 0.7491 and RMSE at 0.0284. Junttila et al. (2022) developed a normalized vegetation index $\left(R_{1390}-R_{1370})/(R_{1390}+R_{1370}\right)$ to estimate leaf water content, achieving an R² of 0.96 and RMSE of 0.0341. Yang et al. (2023a) computed three types of vegetation indices: the empirical vegetation index, he random combination dual-band vegetation index, and the ‘trilateral’parameter, and correlated them with the leaf water content (LWC) and equivalent water thickness (EWT) of camphor trees. Different vegetation indices were used as inputs to build various models for estimating the LWC and EWT of Cinnamomum camphora. The results indicated that the RF model is the best model for estimating LWC and EWT. For the LWC estimation model, the inputs include $R_{900}/R_{970}$ , $\left(1+0.16)(R_{800}-R_{670})/(R_{800}+R_{670}+0.16\right)$ , $R_{734}-R_{956}$ , $FDR_{1009}-FDR_{774}$ , and red-edge amplitude( Dr ), with R² and RMSE values of 0.848 and 0.0057, respectively; For the EWT estimation model, the inputs are $\left(R_{700}-R_{1167})/(R_{700}+R_{1167}\right)$ , $R_{860}/R_{1240}$ , $R_{700}-R_{1167}$ , $FDR_{1182}-FDR_{1514}$ , and red-edge area ( SDr ), with R² and RMSE values of 0.887 and 0.0006, respectively. In the aforementioned studies, scholars have mostly focused on constructing leaf water content estimation models for individual species, with a lack of validation regarding the models’ generalization capabilities across different species.

Some scholars have established cross-species models for estimating plant leaf water content across multiple plant species. Champagne et al. (2003) acquired hyperspectral data for Bean, Canola Corn, Pea and Wheat across the spectral range of 440–2500 nm. They applied a physical model incorporating spectral matching technology to the hyperspectral dataset and directly computed the canopy equivalent water thickness (EWT) via the lookup table method. When analyzing all crop types collectively, the model demonstrated a relatively high predictive accuracy for water content, achieving a consistency index (D) of 0.92 and a root - mean - square error (RMSE) equivalent to 26.8% of the mean. However, the predictive accuracy for wheat alone was notably lower, with an RMSE of 69.9%. Upon excluding wheat from the analysis, the RMSE decreased to 1.79%, while D increased to 0.87. Jones et al. (2004) collected hyperspectral data from 300 to 2400 nm for three crops, corn, spinach, and snap bean in a greenhouse and calculated the average reflectance in specific bands (960 ± 10 nm, 1150–1260 nm, 1450 ± 10 nm, 1950 ± 10 nm, and 2250 ± 10nm) and correlations between five common vegetation indices and water content. The most accurate estimates of water content for corn and snap bean had r^2^ values of 0.67 and 0.50, respectively, while spinach had an r^2^ value of 0.94. It can be seen that these studies mostly focus on herbaceous plants such as corn, wheat, and legumes, with fewer studies on woody plants and across different growth forms.

In summary, most existing studies focus on single-species research and cross-species research on herbaceous plants, with a lack of models for estimating leaf water content in woody plants across species and validation across different growth forms.

To address the above issues, this study selected the peach tree (*Prunus persica* (L.) Batsch), a woody plant of the Rosaceae family and Prunus genus, as the modeling material. For model validation, apple tree (*Malus pumila Mill.*), a congeneric but different-genus species within Rosaceae, and romaine lettuce (*Lactuca sativa* var. *asparagina* L.H.Bailey ex Holub), a herbaceous plant, were chosen. The research objectives are as follows:

1. Acquire hyperspectral data and water content measurements of peach tree leaves. Develop leaf water content retrieval models using two approaches, namely “vegetation index construction” and “feature wavelength selection,” and determine the optimal model through comparative analysis;
2. Apply the optimal model to the reflectance values of each pixel in the leaf hyperspectral images to obtain predicted water content values, thereby enabling the visualization of leaf water distribution;
3. Collect hyperspectral data and water content measurements from apple tree leaves (a woody plant) and romaine lettuce leaves (a herbaceous plant) to validate the generalizability of the developed model both within the same growth type and across different growth types.

## 2 Materials and methods

### 2.1 Technical route

**Figure 1:**
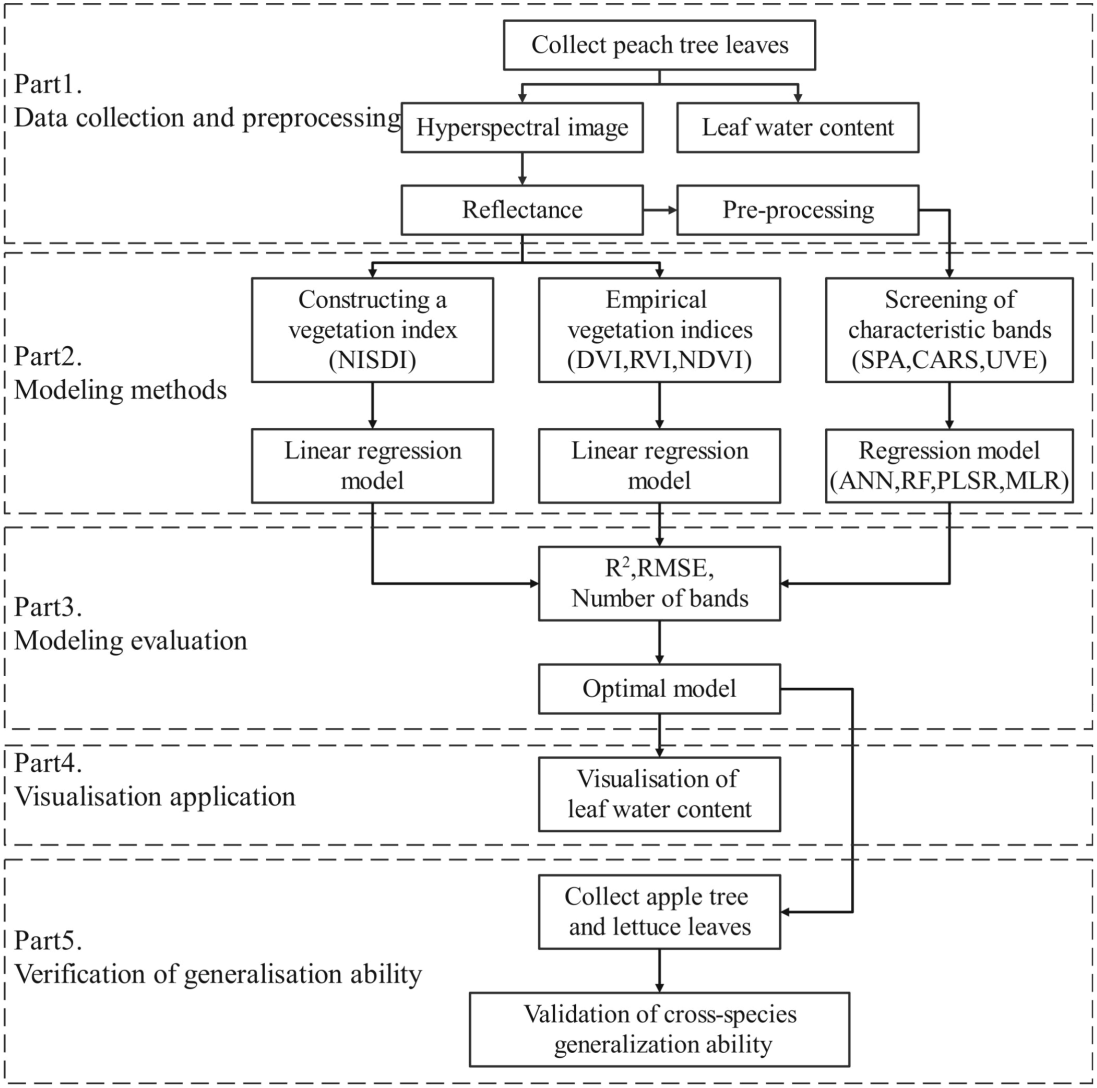
Technical route.

The technical route of this study is illustrated in **Figure 1**. It follows the framework of “data collection and preprocessing - modeling methods - model evaluation - visualization application - generalization ability validation.” Initially, the water content and hyperspectral data of peach tree leaves were collected, and the reflectance data were extracted and preprocessed. Subsequently, leaf water content regression models were established through the construction of vegetation indices, empirical vegetation indices, and selection of characteristic wavelengths. The models were evaluated comprehensively based on the evaluation indicators R2, RMSE, and the number of wavelengths used in modeling, and the optimal model was selected. The reflectance of each pixel in the hyperspectral image was imported into the optimal model to predict the water content of each pixel, thereby achieving the visualization of leaf water content. Finally, the water content and hyperspectral data of apple tree leaves and lettuce leaves were utilized to validate the generalization ability of the optimal model.

### 2.2 Data collection

#### 2.2.1 Leaf collection

##### 2.2.1.1 Peach tree leaves collection

The collection of peach tree leaves took place at the orchard of Nongfeng Agricultural Technology Co., Ltd., located in the Luoyang High-Tech Zone, Henan Province, with geographical coordinates at 112°31′E, 34°58′N. The region experiences a warm temperate continental monsoon climate. The probability of still wind occurrence is 0.6%. The annual average wind speed, excluding still winds, is 3.3 m/s, while the annual average wind speed without excluding still winds is 3.2 m/s. The dominant wind direction is westerly. During spring and summer, the prevailing wind is northeasterly with an average wind speed of 3.1 m/s. In autumn, the prevailing wind is northeasterly with an average wind speed of 3.2 m/s. In winter, the prevailing wind is westerly with an average wind speed of 3.1 m/s. The average annual temperature ranges from 12.2 to 24.6°C, and the frost-free period exceeds 210 days. Annual precipitation varies between 528 and 800 mm. The annual sunshine duration is between 2200 and 2300 hours, and the average annual humidity is 60 - 70%. The management of irrigation and fertilization follows the local average standards, and the cultivar used in the experiment is “Yan Zhi “ crisp peaches. The peach trees have an approximate height of 2.6 m and a trunk diameter of about 7.5 cm. The row spacing is roughly 3.5 m, while the spacing between individual plants is around 3 m. The data was collected on April 24, 2024 (during the new shoot growth stage) and May 27, 2024 (during the fruit enlargement stage). Seventy peach trees with intact canopies and vigorous growth were chosen for sampling. To improve the study’s generalizability and to obtain leaves with diverse water contents, the canopies of the peach trees were divided into upper, middle, and lower sections. In each section, one healthy and intact leaf was randomly collected from each of the four cardinal directions (east, south, west, and north), with the four leaves forming a single group. A total of 420 sets of peach tree leaves were collected across the two sampling events. The collected leaves were sealed, labeled (e.g., the upper leaves from the 36th peach tree in the first collection were labeled 1-36u; the middle leaves from the 40th peach tree in the first collection were labeled 1-40m; the lower leaves from the 52nd peach tree in the second collection were labeled 2-52l), placed into an insulated box containing ice packs and stored in a dark environment to preserve them for subsequent hyperspectral and water content data collection.

##### 2.2.1.2 Cross-species leaves collection

To evaluate the generalization ability of the model and confirm its accuracy and reliability across different species, this study collected two types of cross-species leaves: apple tree leaves and lettuce leaves, to validate the optimal model.

Apple tree leaves were collected from the high-standard water-saving irrigation experimental field at the Xiyuan Campus of Henan University of Science and Technology in Luoyang City, Henan Province. The geographical location is 112°21′E, 34°39′N, characterized by a warm temperate continental monsoon climate, with an average annual temperature of 15.8°C and an average annual precipitation of 578.2 mm. The prevailing wind direction in the experimental area is consistent with the prevailing wind direction in the peach tree picking area. The test material consisted of 5-year-old Yanfu No. 8 apple trees, grafted onto Pingle Sweet Tea rootstock. The apple trees have an average height of approximately 1.7 m and a stem diameter of about 3.5 cm. The spacing between rows is roughly 2.5 m, while the spacing between individual plants is around 2 m. The collection date was October 17, 2024 (during fruit maturity). A total of 61 healthy and intact leaves were randomly collected, numbered, and stored in an insulated box with ice packs. The methods for collecting water content and hyperspectral data were identical to those used for peach tree leaves. A total of 244 apple tree sample datasets were obtained.

Lettuce was purchased from DaZhang Supermarket in JianXi District, Luoyang City, Henan Province, on November 20, 2024. The lettuce leaf is composed of a large leaf vein and surrounding leaf tissue. Given the large size of lettuce leaves, intact leaves are susceptible to damage during the experimental process. In this experiment, a region of approximately 5 cm × 10 cm, which is relatively flat, was randomly selected as the sample. A total of 61 samples were collected, of which 38 samples contained only leaf tissue, while the remaining 23 samples included both leaf veins and leaf tissue. The samples were numbered and stored in an insulated box with ice packs. The methods for collecting water content and hyperspectral data were the same as those used for peach tree leaves. A total of 244 lettuce sample datasets were obtained.

#### 2.2.2 Leaf water content measurement

After data collection was completed, the collected samples were brought back within 30 minutes. The mass of each group of leaves was measured using an electronic scale (accuracy 0.01g), denoted as G_1_, and hyperspectral images were taken. Then, the leaves were dried in an electric thermostatic incubator at 50°C for 55 minutes. After drying, the leaves were taken out, cooled to room temperature (approximately 20 to 25 minutes), and weighed again, denoted as G_2_, and hyperspectral images were taken again. This process was repeated 3 times, and the masses of the leaves were denoted as G_3_ and G_4_, with hyperspectral images taken each time. Finally, the leaves were dried at 85°C until a constant weight was reached, denoted as G_0_. The water content calculation formula is shown in Equation 1.


(1) $$M_{n}=\frac{G_{n}-G_{0}}{G_{n}}\times 100\%$$

Where, $M_{n}$ is the water content of the leaves measured at the nth time, $G_{n}$ is the mass measured at the nth time (g), and $G_{0}$ is the dry mass of the leaves (g), with *n* ranging from 1 to 4. The statistical conditions of leaf water content for each type of sample are presented in **Table 1**.

**Table 1:** The statistical conditions of leaf water content for each type of sample.

Sample type	Dataset	Sample size	Maximum	Minimum	Mean	Variance
Peach tree	all	1597	0.7661	0.1429	0.4767	0.0347
	Modeling Set	1118	0.7661	0.1429	0.4774	0.0348
	Testing Set	479	0.7500	0.0200	0.4751	0.0346
Apple tree	all	244	0.6498	0.0374	0.3942	0.0303
Lettuce	all	244	0.9610	0.0976	0.8395	0.0145
	Without Veins	152	0.9468	0.0976	0.8152	0.0186
	With Veins	92	0.9610	0.5811	0.8796	0.0052

#### 2.2.3 Hyperspectral image acquisition and processing

**Figure 2:**
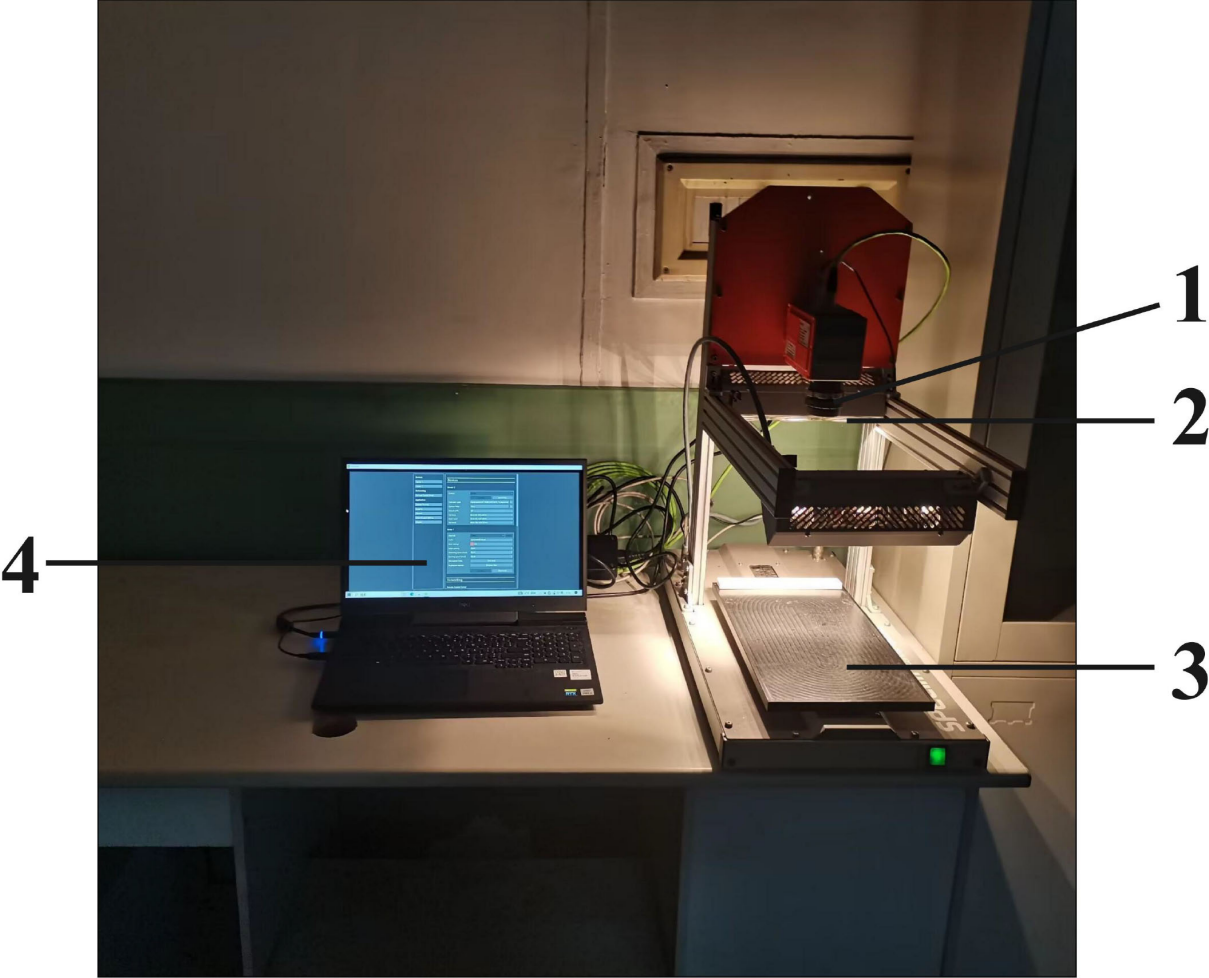
Hyperspectral imaging system. 1:hyperspectral imager, 2:light source, 3:self-stabilizing scanning platform, 4:computer.

The hyperspectral data acquisition system is depicted in **Figure 2**, which includes a hyperspectral imager (SPECIM FX17e, Specim, Finland); a self-stabilizing scanning platform (SPECIM Lab Scanner 40×20cm); two sets of 150W halogen lamp array light sources, optical fibers, and a computer. The hyperspectral imager has a wavelength range of 900–1700 nm, with 224 wavelengths, a sampling interval of 3.5 nm, and an optical resolution of 8 nm. Prior to hyperspectral image acquisition, the leaf surface was cleaned with lens paper to remove dust, and the hyperspectral imager was preheated for 30 minutes to enhance image stability. During acquisition, to avoid the influence of dark current and unstable light sources, the curtains should be drawn, and the lights should be turned off to ensure that the acquisition process takes place in a dark environment. The camera exposure time was set to 6.5 ms, the frame rate to 50 Hz, and the platform moving speed to 20 mm/s. The hyperspectral data were collected using the Lumo Scanner software (Specim, Finland).

To mitigate the effects of illumination and detector sensitivity on the image quality, the hyperspectral images obtained were subjected to black and white calibration. A standard calibration plate with 99% reflectance served as the white reference, while the dark current was captured with the lens cap on to serve as the black reference. The black and white calibration was carried out based on Equation 2:


(2) $$R=\frac{I-B}{W-B}$$

**Figure 3:**
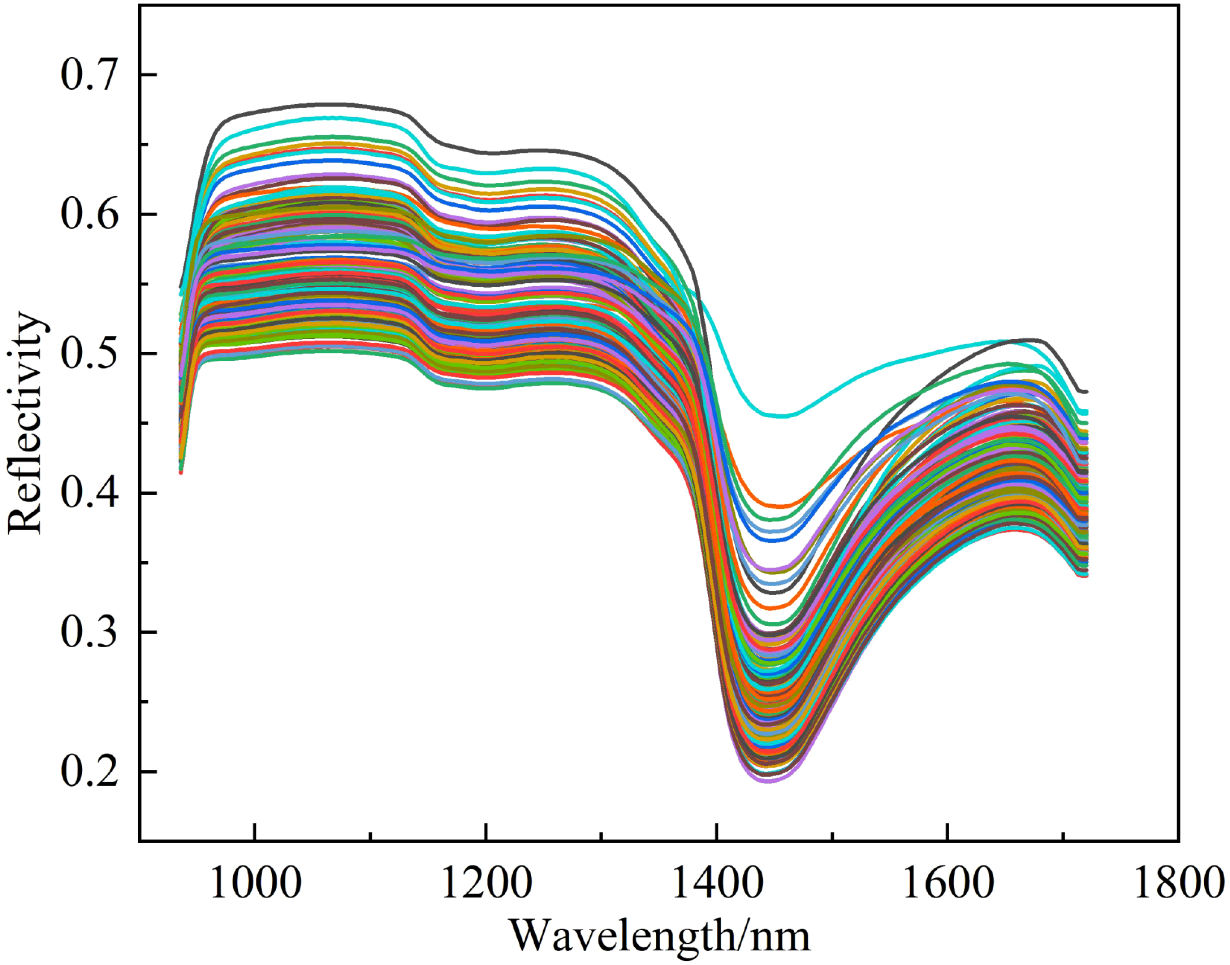
Spectral reflectance curve of peach tree leaves.

Where, *R* represents the corrected spectral reflectance, *I* represents the original spectral reflectance, *W* is the white reference, and *B* is the black reference. The corrected hyperspectral data includes two categories: peach tree leaves and background. Thus, Support Vector Machine Classification in ENVI5.6 (Harris Geospatial Solutions, USA) was employed for supervised classification of leaves and background to eliminate the irrelevant background data. The average reflectance of all pixels in the leaf region was computed as the reflectance data for that sample group, which will be utilized for subsequent data analysis and modeling. The spectral reflectance curve of the peach tree leaf samples is illustrated in **Figure 3**.

### 2.3 Estimation of leaf water content based on vegetation indices

Vegetation indices are dimensionless constants derived from mathematical operations on the reflectance of objects in two or more different wavelength ranges. These indices can enhance certain characteristics or details of vegetation (Gao, 1995), and provide information on the coverage and growth status of vegetation on the ground. In this study, we constructed vegetation indices and three empirical vegetation indices were used as inputs. Their abilities to estimate leaf water content were evaluated through linear regression models.

#### 2.3.1 Vegetation indices construction

Water molecules (chemical formula H_2_O) contain two O-H covalent bonds. The different forms of O-H bond stretching vibrations produce multiple vibrational energy levels. The energy differences between these levels can absorb photons in the near-infrared spectral region, especially in the first overtone region from 1300nm to 1600nm, where there are multiple water absorption peaks (Wang and Yang, 2017; Xu et al., 2011). Among them, the absorption peak around 1440nm is particularly strong. Based on the position of the absorption peak and its derived near-infrared water vegetation index, the water content in vegetation leaves can be reflected. Through the quantitative relationship between them, a vegetation leaf water content estimation model based on the near-infrared vegetation index can be constructed, thereby enabling rapid and accurate estimation of leaf water content. Based on the absorption characteristics of water molecules in the near-infrared region, this study proposes a new vegetation index: Near-Infrared Slope Difference Index (NISDI). The specific calculation equation is shown in Equation 3.


(3) $$NISDI=1.3\times 10^{3}D_{1559}-1.9R_{1439}$$

Where, *D* denotes the slope, which is the rate of change in reflectance between adjacent wavelengths; *R* denotes reflectance.

#### 2.3.2 Empirical vegetation indices

Combinations of wavelengths within the 900–1700 nm range were used to create various difference vegetation indices (Difference Vegetation Index, DVI), ratio vegetation indices (Ratio Vegetation Index, RVI), and normalized difference vegetation indices (Normalized Difference Vegetation Index, NDVI) (Sun et al., 2019). The equations are shown in Equations 4–6. The correlation coefficient r between these vegetation indices and water content was computed, and the wavelength combination yielding the highest absolute value of r was chosen as the new vegetation index.


(4) DVI=NIR−Red

(5) RVI=NIR/Red

(6) NDVI=(NIR−Red)/(NIR+Red)

Where, NIR and Red denote the reflectance in the near-infrared and red bands, respectively.

#### 2.3.3 Modeling methods

The Pearson correlation coefficient measures the strength of the linear relationship between two variables. The closer its absolute value|*r*| is to 1, the stronger the relationship between the variables. A two-tailed significance test (p-value) is performed on the calculated correlation coefficient to determine whether the correlation is significant. The smaller the p-value, the more significant the correlation. A p-value less than 0.01 indicates that the correlation is significant at the 0.01 level, while a p - value less than 0.05 indicates that the correlation is significant at the 0.05 level. In this study, IBM SPSS Statistics 27 was used to calculate the Pearson correlation coefficients between NISDI, DVI, RVI, NDVI, and leaf water content. If a significant linear correlation exists between vegetation indices and water content, linear regression models are established using vegetation indices as inputs.

### 2.4 Estimation of leaf water content based on characteristic wavelengths

#### 2.4.1 Reflectance data preprocessing

Hyperspectral data collection is prone to interference from environmental and instrumental factors, resulting in noise, baseline drift, and spectral variability. To mitigate the impact of these issues on data quality and accuracy, this research utilized Standard Normal Variate (SNV) (Barnes et al., 1989) for the preprocessing of hyperspectral data. By performing Gaussian normalization on the data, spectral discrepancies due to sample particle size, uneven distribution, or lighting conditions are mitigated, ensuring the comparability of reflectance across different samples at equivalent wavelengths. Furthermore, Savitzky-Golay smoothing (SG) (Savitzky and Golay, 1964), normalization (Nor) (Chu et al., 2004), multiplicative scatter correction (MSC) (Geladi et al., 1985), and first derivative (FD) (Norris and Hart, 1996)were employed as comparative preprocessing techniques. To evaluate the efficacy of various preprocessing approaches, the original spectral data and the data post 9 distinct preprocessing methods (SG, Nor, SNV, MSC, FD, SG+Nor, SG+SNV, SG+MSC, SG+FD) were inputted into Partial Least Squares Regression (PLSR) models, with leaf water content as the dependent variable. The optimal number of principal components was ascertained through 10-fold cross-validation, and model performance was evaluated using a validation set. This process ultimately identified the most effective spectral preprocessing technique. The aforementioned data preprocessing methods were all implemented using The Unscrambler X 10.4 (CAMO Software AS, Norway).

#### 2.4.2 Characteristic wavelength selection

Given the vast amount of hyperspectral data and the pronounced multicollinearity among the data, to mitigate redundant information in hyperspectral data and avert model overfitting, the Competitive Adaptive Reweighted Sampling (CARS) algorithm (Li et al., 2009) was utilized. This approach incrementally eliminates irrelevant variables to preserve informative ones, thereby reducing redundancy in hyperspectral data and accomplishing dimensionality reduction. Concurrently, the Successive Projections Algorithm (SPA) (Araújo et al., 2001) and Uninformative Variable Elimination (UVE) (Centner et al., 1996) were implemented as comparative methods for feature wavelength selection from the preprocessed full-spectrum data. The CARS algorithm ascertained the quantity of feature wavelengths to retain based on the minimum root mean square error cross-validation (RMSECV) throughout the iterative process, whereas the SPA and UVE algorithms identified the feature wavelength count by calculating the minimum root mean square error across varying numbers of variables. The CARS algorithm was conFigured with 50 Monte Carlo sampling iterations and employed a 10-fold cross-validation approach for feature wavelength selection, with the SPA algorithm capping the maximum number of wavelengths at 30.The aforementioned characteristic wavelength selection methods were implemented using VS Code (Microsoft, USA) and Python 3.8.

#### 2.4.3 Modeling methods

In this research, three feature wavelength selection results and full-spectrum data were used as inputs to construct a Random Forest (RF) model (Breiman, 2001). Comparative methods included Partial Least Squares Regression (PLSR) (Geladi and Kowalski, 1986), Multiple Linear Regression (MLR) (Jobson and Jobson, 1991), and Artificial Neural Network (ANN) (McCulloch and Pitts, 1943). Experimentation determined that the RF model should have 100 decision trees with a random seed set to 42. The PLSR model was conFigured with 7 principal components, while the ANN model featured 4 hidden layers, employed the ReLU activation function, and underwent training for 1000 iterations. The modeling processes for RF and ANN were carried out using VS Code and Python 3.8, whereas the modeling processes for PLSR and MLR were conducted using The Unscrambler X 10.4.

### 2.5 Model evaluation methods

In this study, the precision of the models was evaluated using two metrics: the Coefficient of Determination (R^2^) and the Root Mean Square Error (RMSE). R^2^ reflects the degree of fit between the predicted and actual values; the closer R^2^ is to 1, the better the model fit and the higher the precision. RMSE indicates the deviation between the predicted and actual values, representing the model’s accuracy; the smaller the RMSE, the higher the model’s estimation precision and stability (Bai et al., 2024). Furthermore, in selecting the optimal model, this study also assessed the comprehensive performance of the models by taking into account the number of wavelengths used in model building.

### 2.6 Peach tree leaf water content visualization

The leaf background was removed using ENVI5.6 software, and the reflectance of each pixel in the selected wavelengths was extracted using VS Code and Python 3.8. These reflectance values were input into the optimal model to obtain the predicted water content for each pixel, enabling the visualization of leaf water content distribution.

### 2.7 Validation of model generalization

To validate the cross-species generalization ability of the optimal model, the hyperspectral reflectance data of apple tree and lettuce leaves were input into the optimal water content estimation model trained on peach tree data to obtain the predicted water content values. The model’s applicability to woody plants (apple trees) and herbaceous plants (lettuce) was evaluated using R² and RMSE.

## 3 Results

### 3.1 Correlation between leaf water content and spectra

**Figure 4:**
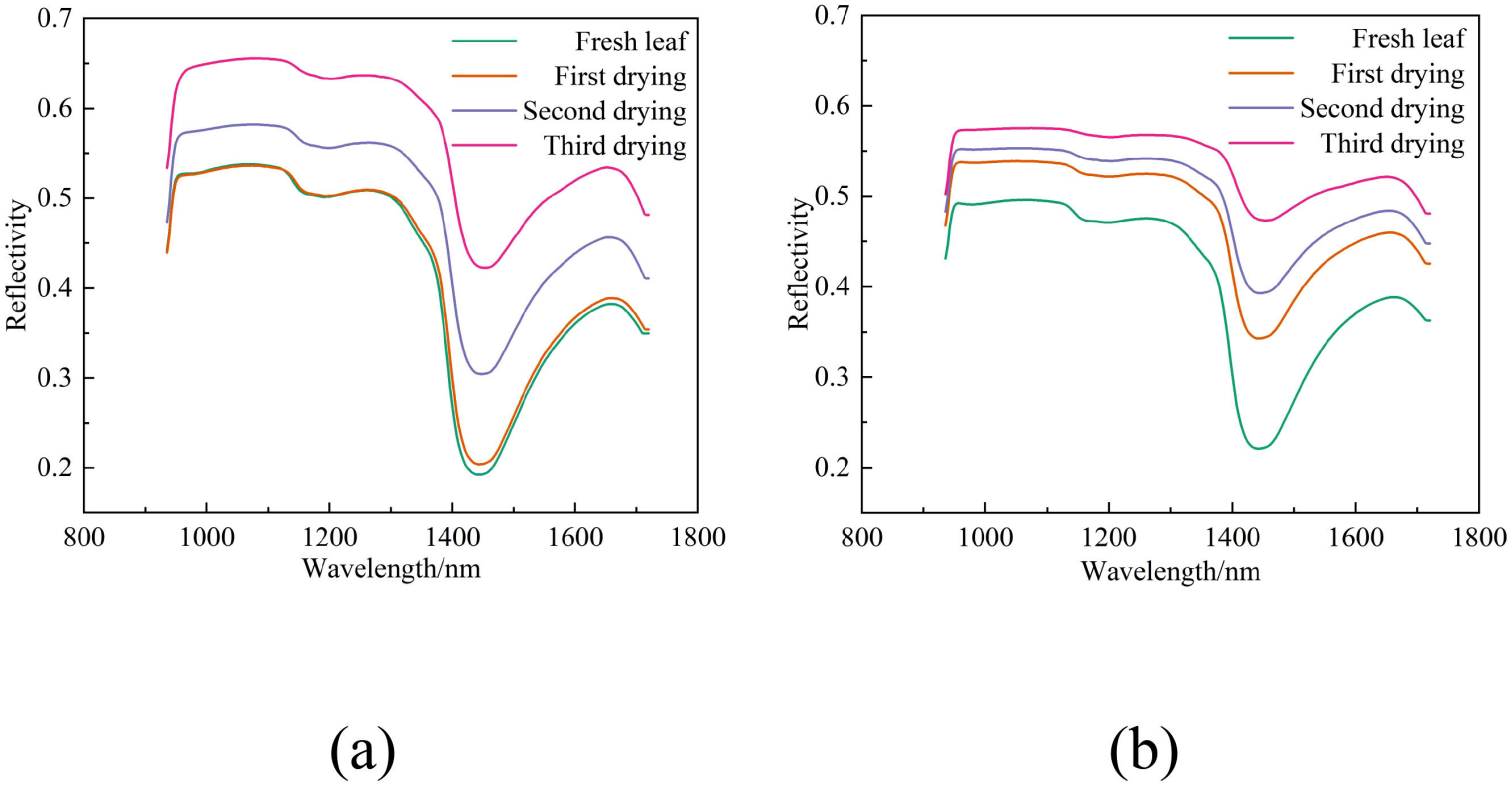
Spectral curves of the same group of leaves under varying water content conditions. **(a)** Spectral curves of leaves labeled 1-36u under four different water content conditions, **(b)** Spectral curves of leaves labeled 2-52l under four different water content conditions.

**Figures 4a, b** display the spectral reflectance curves for two distinct leaf samples at different stages of dehydration. **Figure 4a** illustrates the curves for the upper leaves (labeled 1-36u) from the 36th tree during the initial collection, and **Figure 4b** shows the curves for the lower leaves (labeled 2-52l) from the 52nd tree during the subsequent collection.

Observations from **Figure 4** reveal that as the water content in leaves diminishes throughout the drying process, there is a general upward trend in reflectance, demonstrating an inverse relationship.

**Figure 5:**
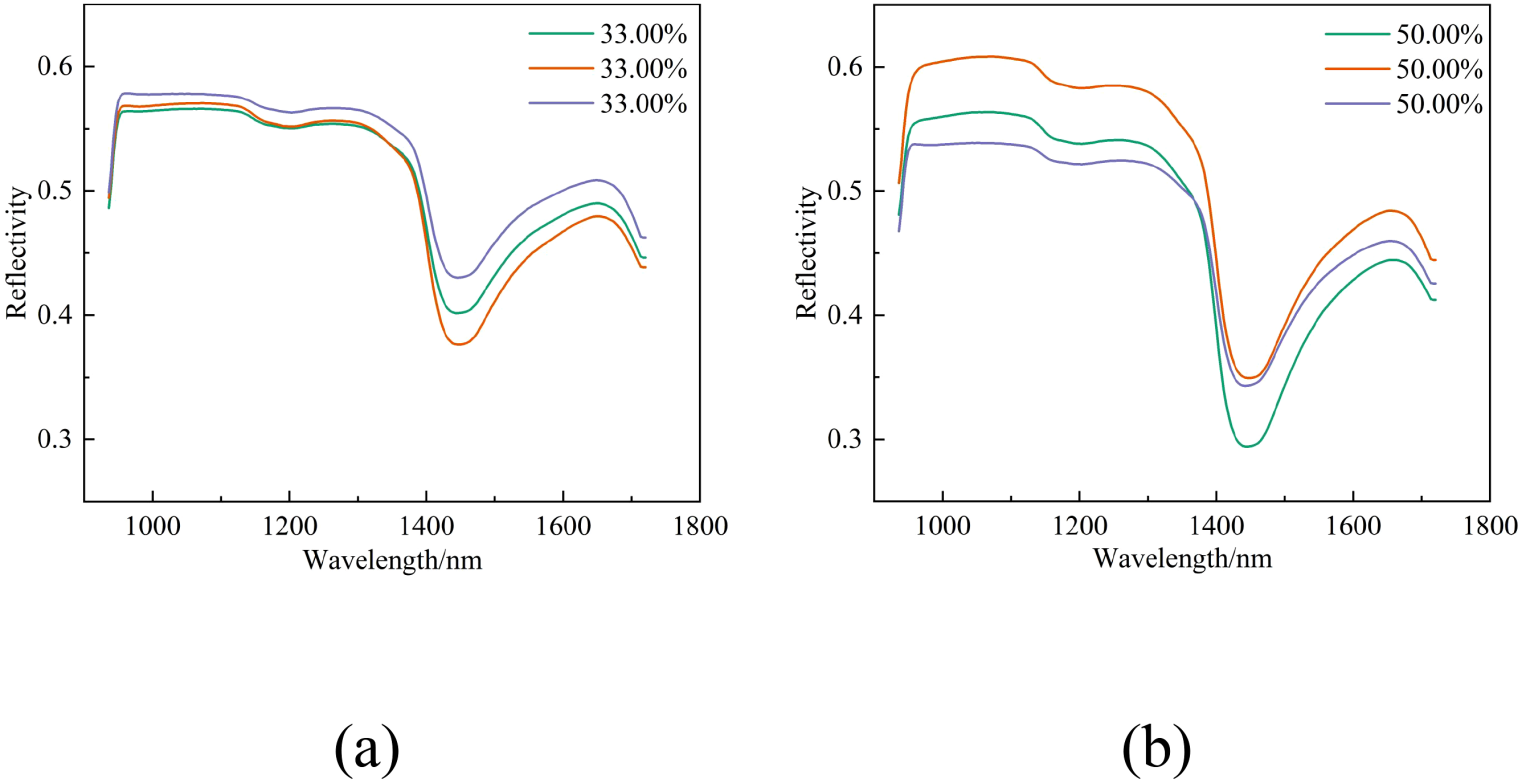
Spectral curves of different groups of leaves under identical water content conditions. **(a)** Spectral curves of leaves with a water content of 33.00%, **(b)** Spectral curves of leaves with a water content of 50.00%.

**Figures 5a, b** depict the spectral reflectance curves for different sets of leaves with water contents of 33.00% and 50.00%, respectively.

As can be seen from **Figure 5**, spectral reflectance varies even when the water content is the same. Consequently, the relationship between different water contents and spectral reflectance is not a straightforward linear one but is subject to the collective influence of multiple variables. To accomplish precise estimation of water content, it is essential to develop and compare a variety of models for analysis.

### 3.2 Estimation results of leaf water content based on vegetation indices

#### 3.2.1 Estimation of leaf water content based on NISDI

**Figure 6:**
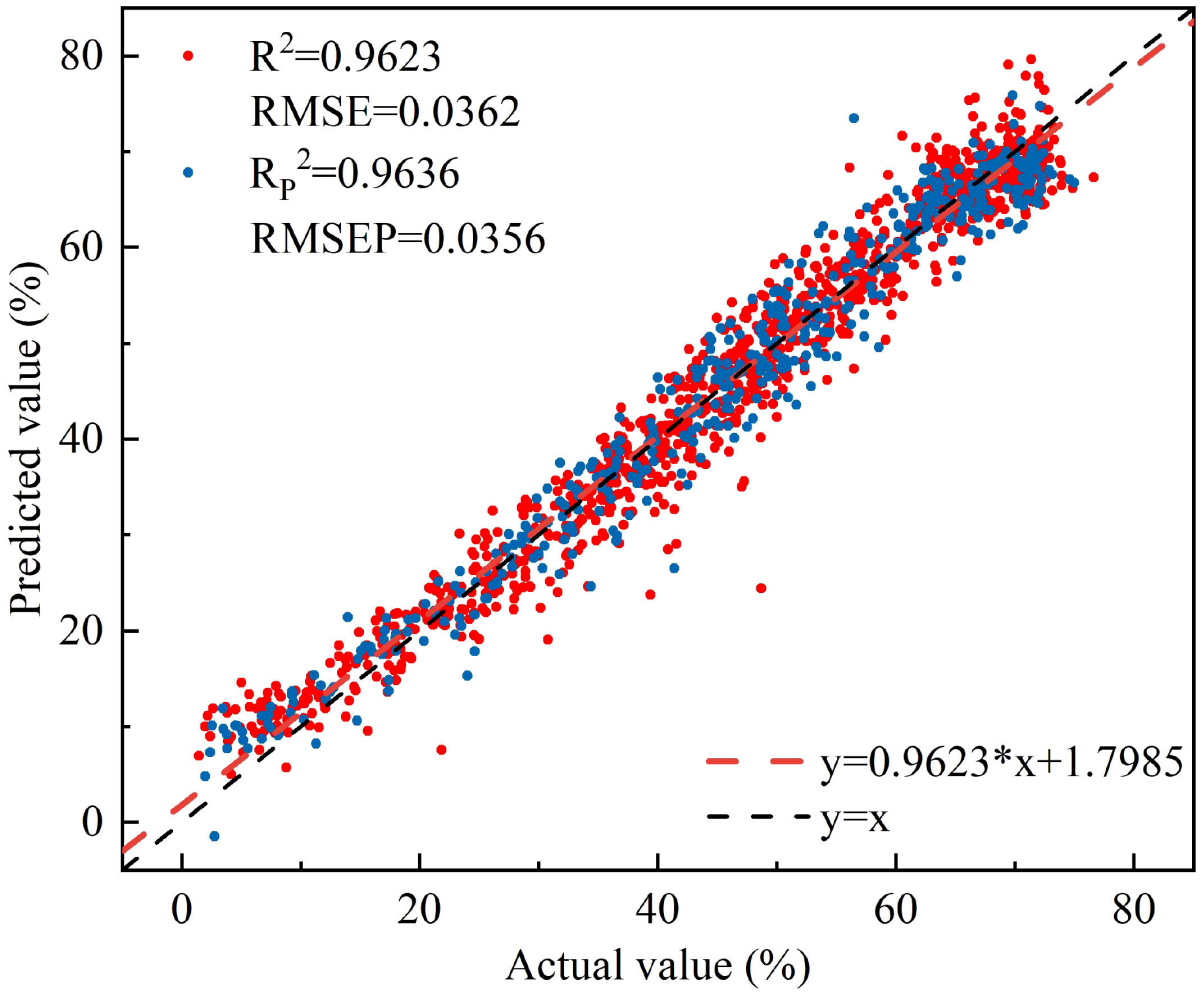
The relationship between the measured and predicted values of peach tree leaves water content based on NISDI.

The Pearson correlation coefficient between the water content of peach tree leaves and NISDI was computed, followed by a significance test. The results indicated a significant correlation between the two variables (r = 0.981, p = 0.000). A linear regression model was developed using the constructed vegetation index NISDI as the input, with the results presented in **Figure 6**.

The model’s R^2^ and RMSE for the calibration set were 0.9623 and 0.0362, respectively, while the R_P_^2^ and RMSEP for the prediction set were 0.9636 and 0.0356, respectively.

#### 3.2.2 Estimation of leaf water content based on empirical vegetation indices

**Figure 7:**
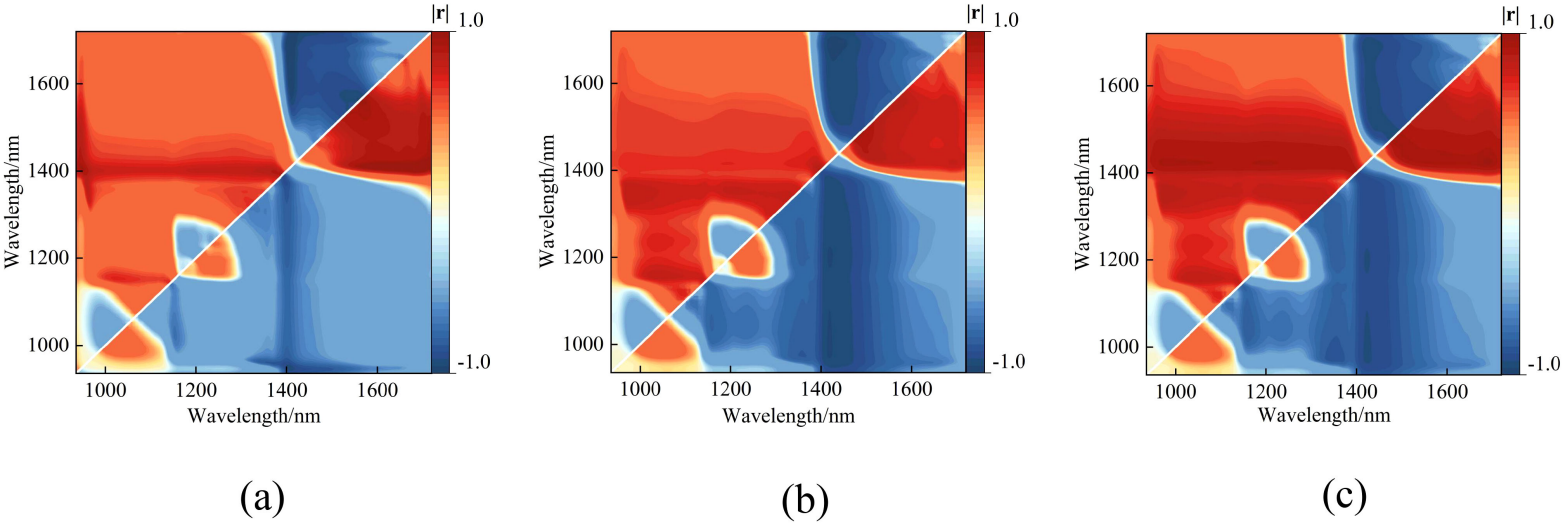
Heatmap of the correlation coefficients between three empirical vegetation indices and the peach tree leaves water content. **(a)** DVI, **(b)** RVI, and **(c)** NDVI.

The original spectral reflectance data were used to readjust the three empirical vegetation indices shown in equations (4) to (6). The correlations between leaf water content and the difference index (DVI), ratio index (RVI), and normalized difference vegetation index (NDVI) calculated from all possible combinations of the 224 wavelengths in the original data were analyzed, with the results shown in **Figure 7**.

The maximum correlation coefficients and corresponding wavelength locations for each vegetation index are presented in **Table 2**.

**Table 2:** The maximum correlation coefficients, p-value and corresponding band positions for the three empirical vegetation indices.

VI	|*r*|*_max_*	p-value	Wavelength Position/*nm*
DVI	0.9796	0.000	(1695,1407)
RVI	0.9806	0.000	(1424,1691)
NDVI	0.9725	0.000	(1691,1425)

**Figure 8:**
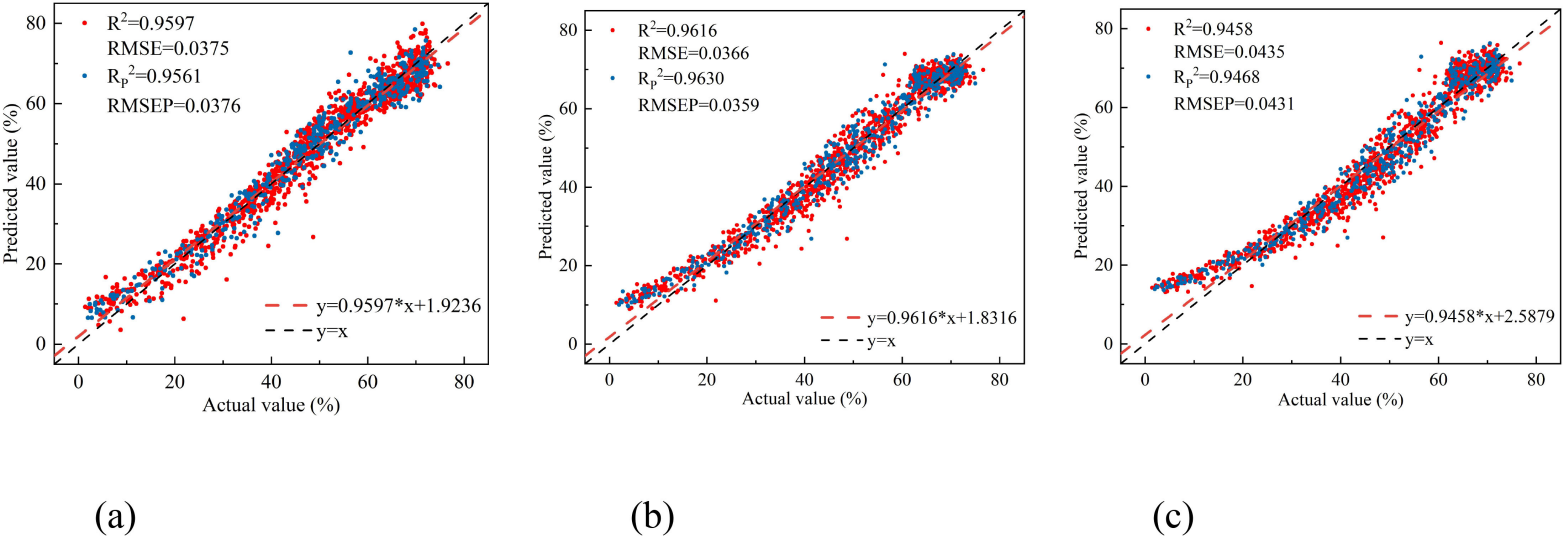
The relationship between the measured and predicted values of peach tree leaves water content based on three empirical vegetation indices. **(a)** DVI(R_1695_,R_1407_), **(b)** RVI(R_1425_,R_1691_), and **(c)** NDVI(R_1691_,R_1425_).

The results show that the vegetation indices DVI (R_1695_, R_1407)_, RVI (R_1425_, R_1691_), and NDVI (R_1691_, R_1425)_ all exhibit significant linear correlations with leaf water content. Linear regression models were developed using the vegetation indices at the band positions corresponding to the maximum correlation coefficients as inputs. The results are shown in **Figure 8**.

Among the three empirical vegetation indices, RVI (R_1425_, R_1691_) performed the best, with an R_P_^2^ of 0.9630 and an RMSEP of 0.0359. DVI (R_1695_, R_1407_) followed closely, with an R_P_^2^ of 0.9561 and an RMSEP of 0.0376. NDVI (R_1691_, R_1425_) had the least favorable results, with an R_P_^2^ of 0.9468 and an RMSEP of 0.0431. Overall, the linear regression models constructed using the four vegetation indices all effectively estimated the leaf water content of peach tree leaves. The linear regression model based on NISDI performed the best, with an R_P_^2^ of 0.9636 and an RMSEP of 0.0356.

### 3.3 Leaf water content estimation model based on characteristic wavelengths

#### 3.3.1 Preprocessing of peach tree leaf reflectance data

**Figure 9:**
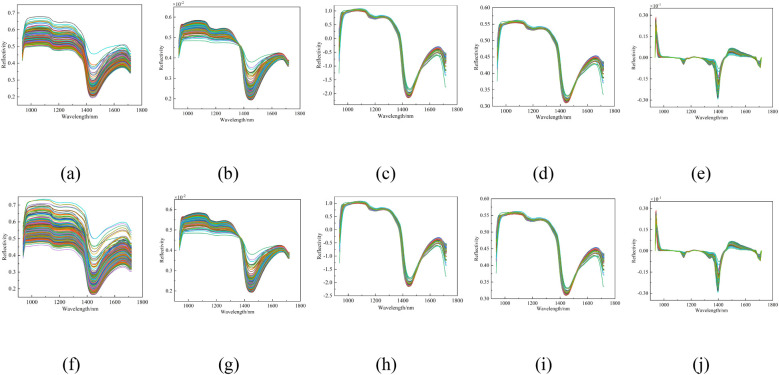
Spectral curves of peach tree leaves after different pre-treatments. **(a)** Original, **(b)** Nor, **(c)** SNV, **(d)** MSC, **(e)** FD, **(f)** SG, **(g)** SG+Nor, **(h)** SG+SNV, **(i)** SG+MSC, and **(j)** SG+FD.

In this study, nine preprocessing methods were applied to the spectral data: Savitzky-Golay (SG), normalization, Standard Normal Variate (SNV), Multiplicative Scatter Correction (MSC), first derivative (FD), SG + normalization, SG + SNV, SG + MSC, and SG + FD. The spectral curves after each preprocessing method are shown in **Figure 9**.

The original spectral data, along with the spectral data following nine distinct preprocessing techniques, were employed as inputs to construct a Partial Least Squares Regression (PLSR) model. The results of the modeling are presented in **Table 3**.

**Table 3:** The performance of different preprocessing methods.

Preprocessing Technique		Principal Components	Modeling Set		Validation Set	
			R^2^	RMSE	R_cv_^2^	RMSECV
Original	Original	4	0.9673	0.0337	0.9672	0.0338
	Nor	3	0.9668	0.0339	0.9666	0.0340
	SNV	4	0.9699	0.0323	0.9696	0.0325
	MSC	3	0.9625	0.0361	0.9616	0.0365
	FD	3	0.9625	0.0365	0.9620	0.0363
SG	SG	3	0.9673	0.0339	0.9670	0.0339
	Nor	3	0.9668	0.0339	0.9666	0.0341
	SNV	4	0.9698	0.0325	0.9695	0.0326
	MSC	3	0.9625	0.0361	0.9618	0.0364
	FD	3	0.9625	0.0362	0.9621	0.0363

The modeling results show that the outcomes of different preprocessing methods are quite similar, with all R² values above 0.96. The SNV preprocessing method performs relatively well. For the modeling set, the R² and RMSE are 0.9699 and 0.0323, respectively. For the validation set, the R_cv_² and RMSECV are 0.9696 and 0.0325, respectively. Although the results of SNV preprocessing are only marginally better than those of other methods, SNV is still chosen for subsequent data analysis

#### 3.3.2 Results of characteristic wavelength selection

**Figure 10:**
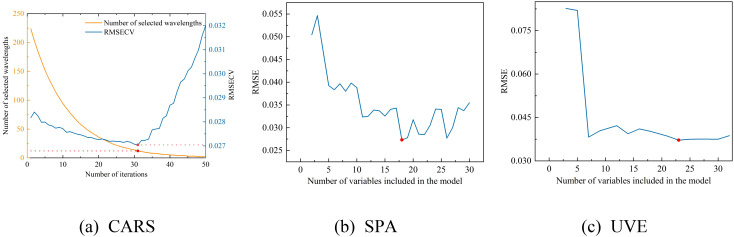
The selection process of the three feature wavelength band screening algorithms. **(a)** the CARS selection process, **(b)** the SPA selection process, and **(c)** the UVE selection process.

Three characteristic wavelength selection methods—SPA, CARS, and UVE—were employed to select characteristic wavelengths from the reflectance data preprocessed by SNV, with the operation processes of each method depicted in **Figure 10**.

As depicted in **Figure 10**, the RMSECV value for the CARS method exhibits a pattern of decreasing and then increasing with the number of iterations, achieving its minimum value of 0.0270 at the 31st iteration,.at which point the CARS method has selected 12 bands. In the case of the SPA method, the RMSE value plummets rapidly and then levels off around 0.030 as the number of variables in the model increases, with the lowest RMSE value recorded at 0.0273, corresponding to 18 selected bands by the SPA method. For the UVE method, the RMSE demonstrates a sharp initial decrease followed by a gradual decline during the selection process, with the smallest RMSE value reaching 0.0372, at which 23 bands are selected by the UVE method. The results of these three feature band selection methods are presented in **Table 4**.

**Table 4:** Results of different feature band selection algorithms.

Feature Selection Method	The number of selected band	Characteristic wavelengths (*nm*)
SPA	18	935.61, 939.06, 945.98, 970.19, 1206.87, 1322.66, 1364.92, 1393.14, 1410.79, 1428.46, 1453.22, 1520.56, 1584.55, 1670.14, 1688.02, 1702.33. 1709.49, 1720.23
UVE	23	935.61, 939.06, 942.52, 945.98, 1375.50, 1379.02, 1382.55, 1386.08, 1389.61, 1393.14, 1428.46, 1431.99, 1435.53, 1439.07, 1442.60, 1446.14, 1513.46, 1517.01, 1520.56, 1524.11, 1527.66, 1670.14, 1673.72
CARS	12	945.98, 1238.39, 1308.59, 1357.87, 1364.92, 1393.14, 1421.39, 1499.27, 1577.43, 1652.29, 1688.02, 1716.65

After applying the CARS method, the number of wavelengths was reduced from 224 to 12, constituting 5.36% of the total wavelengths; with the SPA method, the number of wavelengths decreased from 224 to 18, which is 8.04% of the total wavelengths; and with the UVE method, the number of wavelengths was reduced from 224 to 23, representing 10.27% of the total wavelengths. The CARS method selected the fewest wavelengths and had the lowest RMSE, followed by SPA, while UVE selected the most wavelengths and had the highest RMSE.

#### 3.3.3 Leaf water content estimation model based on different characteristic wavelengths and modeling methods

Four sets of variables, including the full spectrum and the results from the three feature band selection methods, were used as inputs to construct four regression models: Random Forest (RF), Partial Least Squares Regression (PLSR), Multiple Linear Regression (MLR), and Artificial Neural Network (ANN), in order to identify the most effective method for estimating leaf water content. The modeling results for these different methods are presented in **Table 5**.

**Table 5:** Modeling results for different combinations of feature wavelength bands and regression models.

Feature selection method	Regression model	R^2^	RMSE	R_P_^2^	RMSEP
CARS	ANN	0.9907	0.0180	0.9849	0.0228
	RF	**0.9979**	**0.0085**	**0.9861**	**0.0219**
	PLSR	0.9644	0.0352	0.9614	0.0365
	MLR	0.9831	0.0244	0.9798	0.0259
SPA	ANN	0.9878	0.0204	0.9857	0.0223
	RF	0.9979	0.0086	0.9853	0.0225
	PLSR	0.9699	0.0324	0.9650	0.0349
	MLR	0.9830	0.0245	0.9810	0.0257
UVE	ANN	0.9863	0.0217	0.9828	0.0244
	RF	0.9974	0.0095	0.9842	0.0234
	PLSR	0.9499	0.0418	0.9489	0.0420
	MLR	0.9842	0.0237	0.9820	0.0244
Full Spectrum	ANN	0.9757	0.0290	0.9723	0.0310
	RF	0.9957	0.0123	0.9705	0.0319
	PLSR	0.9665	0.0341	0.9669	0.0338
	MLR	0.9902	0.0207	0.9834	0.0241

Bolded portions indicate the best performing modeling results.

**Figure 11:**
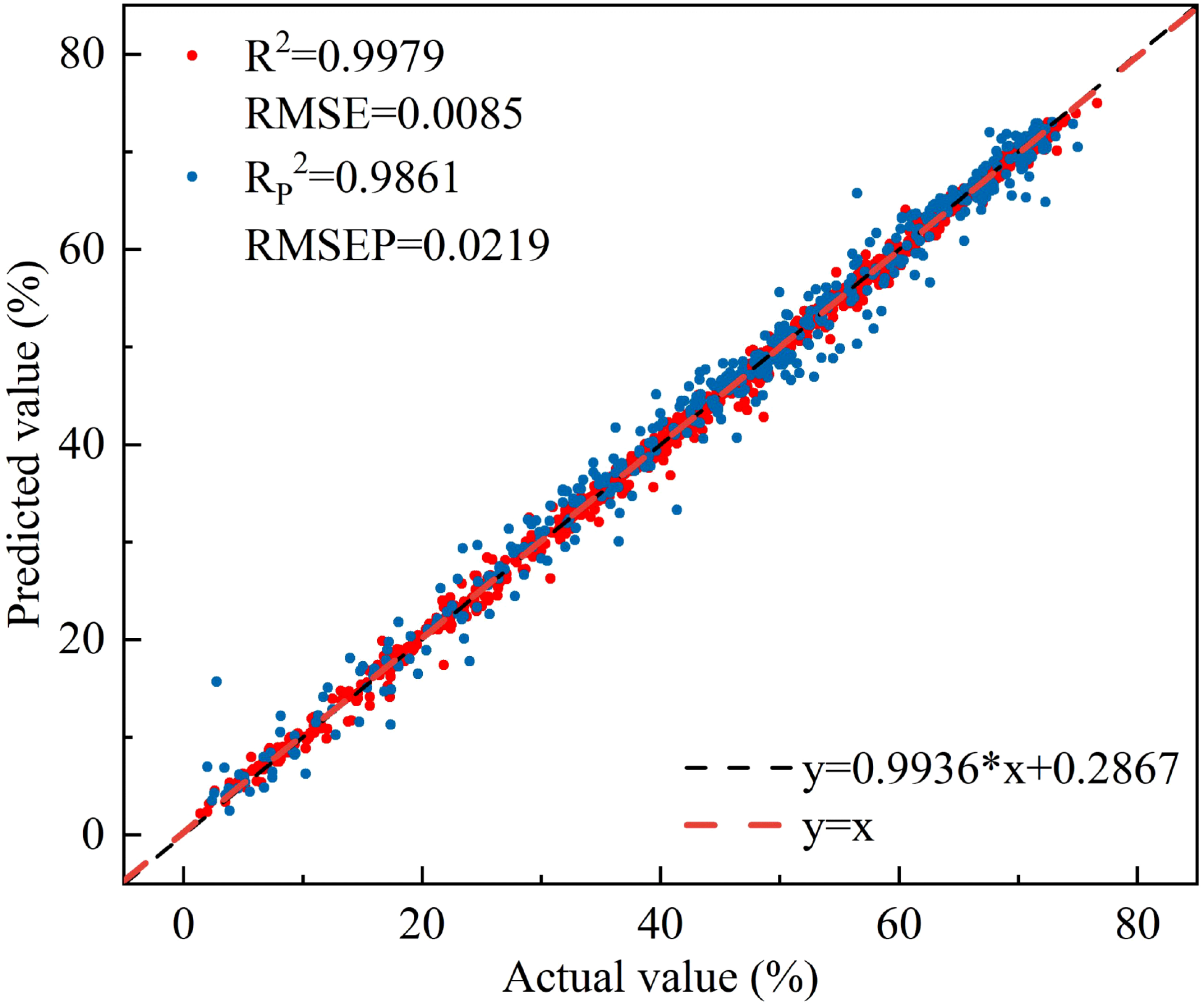
The relationship between the measured and predicted values of peach tree leaves water content based on CARS-RF.

Comparative analysis of different modeling methods reveals that the R^2^ of the ANN and RF models established by each selection method has increased compared with full-spectrum modeling, while the R^2^ of the PLSR and MLR models has decreased. Overall, the R_P_^2^ of each model is greater than 0.90, indicating that each model can effectively estimate the leaf water content of peach trees. There is little difference in stability, accuracy, and predictive ability between the models established using the selected characteristic wavelengths and those established using the full spectrum, but the number of variables is greatly reduced. All three characteristic selection methods effectively removed redundant wavelengths and improved model efficiency, among which the CARS-RF model (**Figure 11**) performed the best, with an R_P_^2^ of 0.9861 and an RMSEP of 0.0219.

Compared with the CARS-RF model, which performed best in characteristic wavelength modeling, the linear regression model established using NISDI exhibited a slight decrease in precision (R_P_^2^ decreased by 0.0225, RMSEP increased by 0.0131). However, the number of wavelengths used in the modeling process was significantly reduced, from 12 to 3, representing a 75% reduction. This greatly enhanced the model’s computational efficiency and stability. Although there was a trade-off in precision, the linear regression model established using NISDI showed clear advantages in wavelength selection, computational efficiency, and model stability. Therefore, this model was determined to be the optimal model.

### 3.4 Visualization of peach tree leaf water content

**Figure 12:**
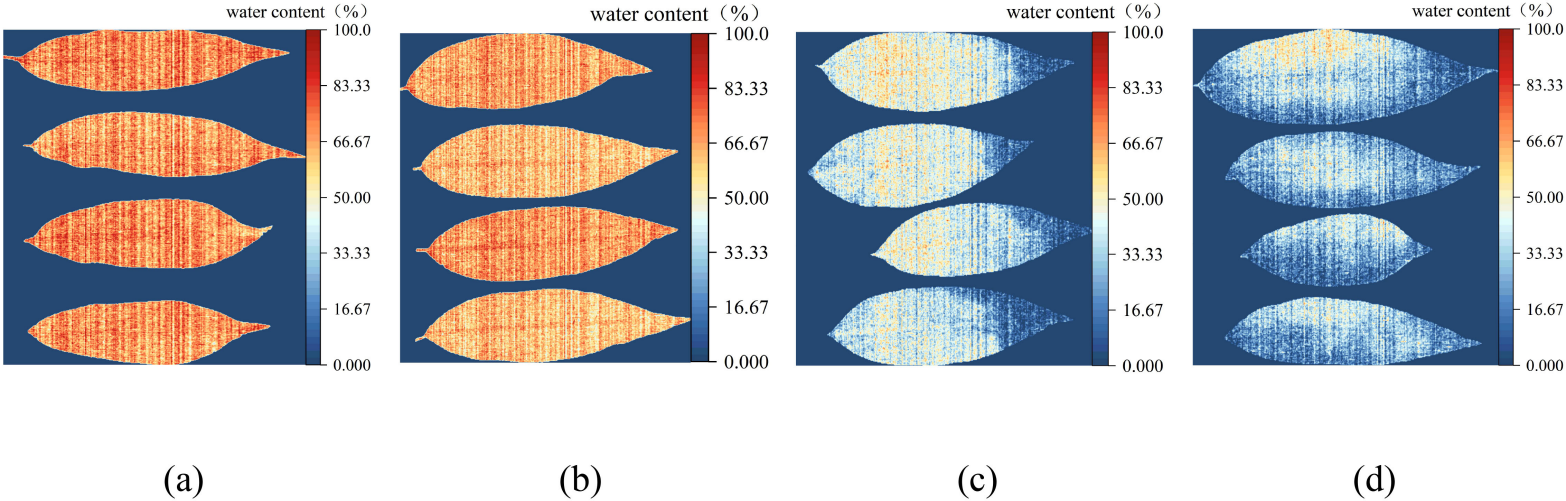
Visualization of the water content of peach tree leaves. **(a)** Water content 76.61%, **(b)** Water content 64.46%, **(c)** Water content 41.41%, and **(d)** Water content 24.43%.

The linear regression model established using NISDI was used to estimate the water content of each pixel in the hyperspectral images of peach tree leaves, achieving visualization of water content. **Figures 12a–d** show the visualization images of peach tree leaf water content with water contents of 76.61%, 64.46%, 41.41%, and 24.43%, respectively.

As can be seen from the figures, the higher the water content, the closer the image is to red. Conversely, the lower the water content, the closer the image is to blue. When the leaf water content is low (as shown in **Figures 12c, d**), the leaf tips and edges appear blue, while the veins appear orange. Overall, the water content in the veins is consistently higher than that in the leaf tissues across each group of leaves. The results indicate that the model is capable of monitoring changes in the water content of peach tree leaves.

### 3.5 Model generalization performance validation

The Pearson correlation coefficients between the water content of apple tree leaves and lettuce leaves and the vegetation index NISDI were calculated, and significance tests were conducted. The results are presented in **Table 6**.

**Table 6:** Correlation coefficients between NISDI and leaf water content for different samples.

Sample type	|*r*|	P-value
Apple leaf samples	0.975	8.283×10^-160^
All lettuce samples	0.707	2.679×10^-38^
Lettuce samples (without veins)	0.906	6.391×10^-58^

**Figure 13:**
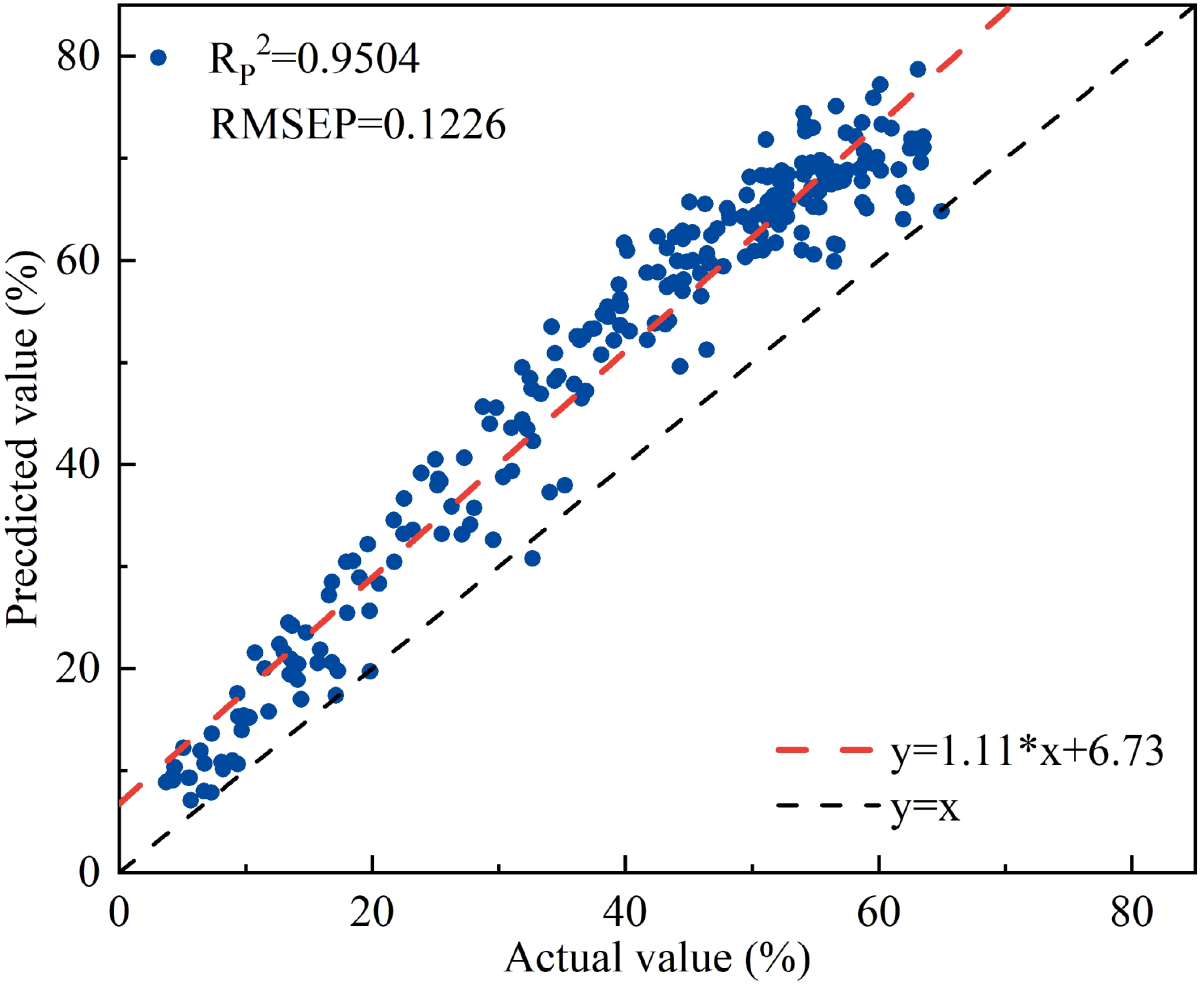
The relationship between the measured and predicted values of apple tree leaf water content.

It was found that the vegetation index NISDI and the corresponding water content of apple leaf samples, all lettuce samples, and lettuce leaf samples all have significant correlations. The water content and reflectance data of apple tree leaves were input into the linear regression model established using NISDI to estimate their water content, with the results presented in **Figure 13**.

The R_P_^2^ and RMSEP were 0.9504 and 0.1226, respectively. Compared with the results for peach trees, the performance of this model is somewhat lower. However, the model still possesses a certain degree of quantitative prediction capability for apple tree leaf water content.

**Figure 14:**
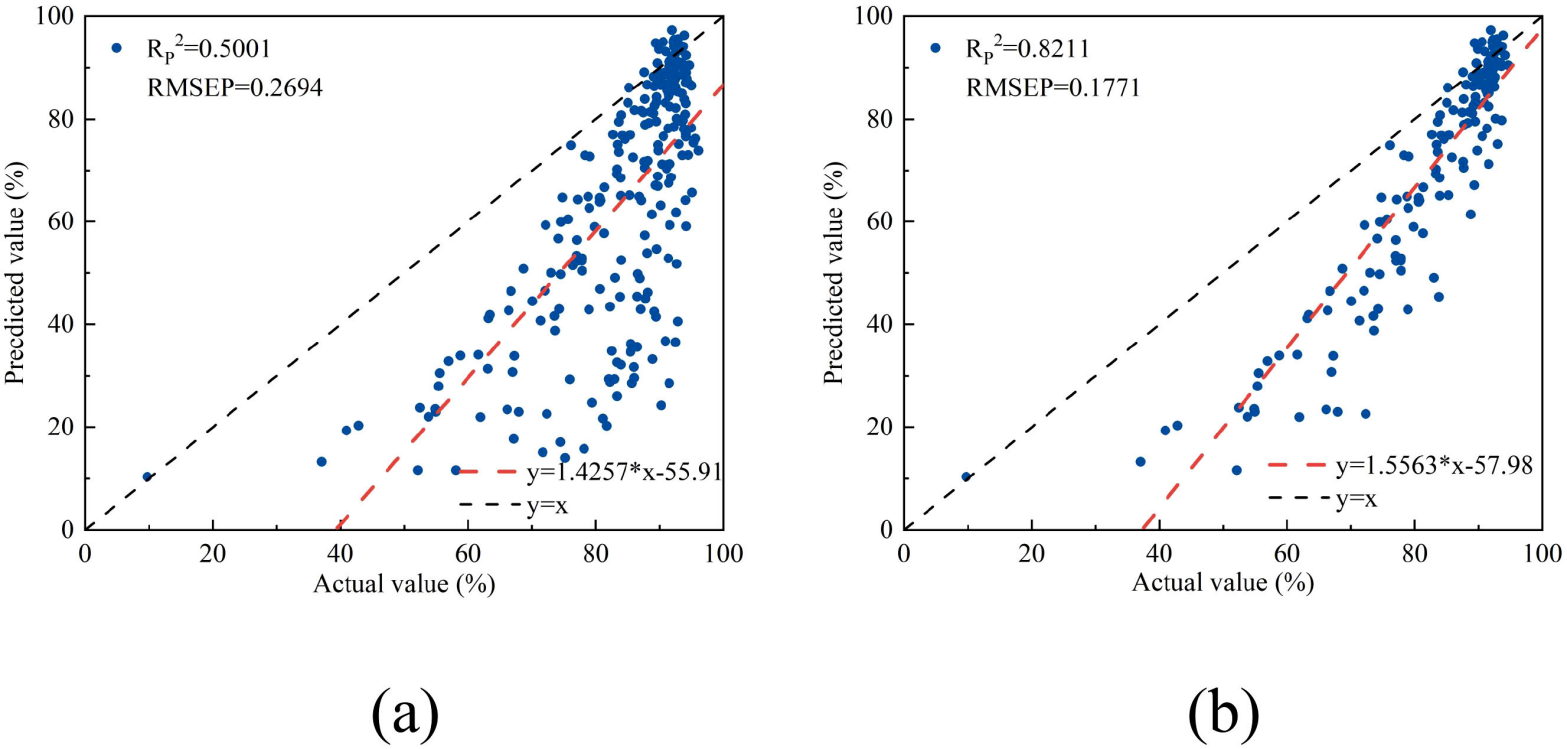
The relationship between the measured and predicted values of water content in chicory leaves. **(a)** all lettuce samples, **(b)** lettuce samples without veins.

The reflectance and water content data of lettuce were input into the linear regression model established using NISDI to estimate their water content, with the results presented in **Figure 14a**. Modeling was conducted separately for the leaves, with the results presented in **Figure 14b**.

For the full dataset of lettuce samples, the modeling results showed an R_P_^2^ of 0.5001 and an RMSEP of 0.2694. When modeling was conducted separately for the leaves, the R_P_^2^ and RMSEP were 0.8211 and 0.1771, respectively. Compared with peach and apple trees, the model performance decreased, indicating a decline in performance when crossing growth forms.

## 4 Discussion

The peach tree (Rosaceae, Prunus) and apple tree (Rosaceae, Malus) selected in this study represent the close-kin differences within the Rosaceae woody plants, while the lettuce (Asteraceae) represents the woody-herbaceous cross-growth form comparison. Both peach and apple tree leaves have typical woody plant characteristics such as a thick cuticle and multiple layers of palisade tissue (Reynoud et al., 2021), but they show significant differentiation in aspects like trichome density. As a herbaceous plant, lettuce has only a single layer of palisade tissue (Luo et al., 2024) and its cuticle thickness is less than half of that of woody plants. These differences in leaf structure provide an ideal gradient for verifying the model’s generalization ability across species. The contributions of this study include: A new vegetation index, the Near-Infrared Slope Difference Index (NISDI), was developed based on the absorption characteristics of water molecules in the near-infrared region. Three types of plant leaves were used as experimental materials to validate the model’s generalization capability within closely related taxonomic units of woody plants (Prunus *vs*. Malus) and to effectively evaluate the model’s performance across different growth forms (woody *vs*. herbaceous).

### 4.1 Correlation analysis between leaf water content and spectral reflectance

Leaf reflectance rises as water content decreases (**Figure 4**). The negative correlation between these two factors may be attributed to the role of water as an optical medium when water content is high. Water fills the intercellular spaces and cells within the leaf, thereby enhancing the leaf’s capacity to scatter and absorb light. This leads to a reduction in the amount of light reflected from the leaf surface and a subsequent decrease in reflectance. In contrast, when the leaf loses water and its water content drops, the intercellular spaces expand, and the leaf tissue’s ability to scatter light diminishes. As a result, more light is reflected from the leaf surface, causing an increase in reflectance. However, even with the same water content, variations in spectral reflectance can be observed (**Figure 5**). These variations may stem from differences in leaf internal structure (Yuwei et al., 2021) and nutritional status, among other factors, that impact reflectance.

### 4.2 Analysis of results from different modeling methods

The modeling results before and after data preprocessing using SG smoothing in this study show little difference. This is because the original hyperspectral data is relatively smooth and does not contain a large amount of noise. The R^2^ of the modeling results using various preprocessing methods are all around 0.96 (**Table 3**). This is because the original data is already in a relatively standard and stable state, so further preprocessing does not significantly change the original data.

The modeling results using characteristic wavelengths (**Table 5**) show that the precision of the ANN and RF models established by each selection method has increased compared with full-spectrum modeling, while the R^2^ of the PLSR and MLR models has decreased. This may be because ANN and RF models are usually more complex and better at handling complex nonlinear relationships. The selected wavelengths remove noise and redundant information, retaining the characteristic wavelengths most related to leaf water content. This allows ANN and RF models to focus more on these relevant pieces of information, no longer being affected by irrelevant or weakly relevant information, thereby improving the model’s precision and generalization ability. In contrast, PLSR and MLR models, which are based on linear relationships for prediction, may not be able to fully capture the true relationship between the remaining characteristics and the target, resulting in lower prediction performance than ANN and RF models.

Yasir et al. (2023) collected hyperspectral data from 277 leaf samples of 10 plant species as the modeling set and used publicly available datasets to validate the modeling results, identifying the three-band vegetation index most suitable for predicting water content. The optimal result had an R^2^ of 0.969 and an RMSE of 0.001, which is better than the precision of the optimal model for peach tree leaves in this study (R_P_^2^ = 0.9636, RMSEP=0.0356). This is because Yasir et al. used leaves from 10 different plant species for modeling, whereas this study only used peach tree leaves for modeling. In the future, more leaves from different plant species should be collected to improve the modeling set. Compared with the previously reported melon canopy leaf water content prediction model (Guo et al., 2022), the number of bands used in this study was reduced by 85%, while R_P_^2^ increased by 0.0604. This phenomenon may be attributed to the fact that the previous study used only 150 samples for modeling, which is a relatively small sample size. Additionally, the reflectance data for each sample was obtained by averaging the values of three points on the leaf. In contrast, this study had a much larger sample size, with 1118 samples in the modeling set. Moreover, the reflectance data for each sample was obtained by averaging the reflectance of all pixel points in the leaf area. Compared with the reflectance extraction method used by Guo et al., the method used in this study provides a more representative reflectance value for the overall sample. Compared with the linear regression model for corn canopy water content constructed by Shu et al. (2022) using two wavelengths (R^2^ of 0.72), the number of wavelengths used in this study increased by one, but the estimation precision of leaf water content for the three types of plant leaves in this study (R_P_^2^ of 0.9636, 0.9504, and 0.8211) is higher than the model proposed by Shu et al. This study further expands the potential of vegetation indices in estimating plant leaf water content.

### 4.3 Analysis of the generalization ability of the optimal model

The optimal model was used to estimate the water content of leaves from the upper, middle, and lower parts of the peach tree, and it was found that the R_P_^2^ was above 0.95 for all (upper: R_P_^2^ = 0.9578, RMSEP=0.0389; middle: R_P_^2^ = 0.9685, RMSEP=0.0330; lower: R_P_^2^ = 0.9658, RMSEP=0.0353).

When the optimal model was used to estimate the water content of apple tree leaves, the R_P_^2^ and RMSEP were 0.9504 and 0.1226, respectively. Compared with peach tree leaves (R_P_^2^ = 0.9636, RMSEP=0.0356), there was a slight decrease in precision (ΔR_P_^2^ = 0.0132, ΔRMSEP=0.0870), but the difference was small. This indicates that the model has good generalization ability for woody plants in the same family, which may be due to the fact that both peach and apple trees are woody plants in the Rosaceae family. They have been long exposed to environmental stresses such as drought and pests, and both have thick cuticles and epicuticular wax as physiological structures (Arya et al., 2021). This similar structure maintains the cross-species consistency of the water-spectrum relationship. However, peach tree leaves have a smooth surface without hairs, while apple tree leaves have short pubescence. This difference may alter the scattering properties of light, resulting in different penetration, reflection, and absorption of near-infrared light in the leaves, which slightly reduces the estimation precision.

Using the optimal model to estimate the water content of lettuce leaves, while the R_P_^2^ remains relatively high, the performance is significantly worse compared to that for peach tree leaves and apple tree leaves. The R_P_^2^ decreased by 0.1425 and 0.1293, respectively. This suggests that the model’s performance has degraded when applied across different growth forms. This may be because lettuce has a short growth cycle and its leaf surface has delicate wax, which is significantly different from the basic structure of the other two types of leaves. Additionally, the model’s ability to estimate the water content of the leaf part of lettuce was stronger than that of the leaf veins. This may be due to the structural differences between the leaf and the leaf veins of lettuce. The leaf is mainly composed of mesophyll cells rich in chloroplasts, while the leaf veins are mainly composed of vascular bundle cells. Another main reason is that the water content of the leaf veins is higher, and the water content of the leaf vein sample set has a smaller degree of dispersion. The maximum water content in the training set is only 76.61%. This mismatch in distribution may prevent the model from effectively generalizing to the extreme data points in the test set, resulting in inaccurate estimation.

### 4.4 Visualization analysis of leaf water content

In this study, when extracting the reflectance of each leaf sample, the average reflectance of all pixel points in the leaf area of each group of samples was calculated as the reflectance data for that group of samples. However, the rich spatial distribution information in the hyperspectral images was not fully explored. To more comprehensively and intuitively demonstrate the significant spatial difference of water content in different regions of peach tree leaves, this study implemented the visualization of leaf water content (**Figure 12**), thereby more intuitively displaying the internal water distribution and the water gradient changes in different parts of the leaf. When the leaf water content is low (as shown in **Figures 12, d**), the leaf tips and edges appear blue, while the veins appear orange, indicating that the leaf tips and edges begin to lose water first. In the four groups of visualization images, the water content of the leaf veins is always higher than that of the leaf tissues. This is because the veins are the main channels and storage structures for water transport and storage, primarily responsible for storing and transporting water. In contrast, although the leaf tissues also contain water, their main function is photosynthesis and gas exchange, with weaker water storage capacity and relatively lower water content (Ellsworth et al., 2023). This phenomenon is consistent with the research results of scholars such as (Sun et al., 2018) and (Liu et al., 2024)

### 4.5 Outlook

This study collected leaves from the economically significant fruit tree, the peach tree, and used leaves from the apple tree, which is in the same family but a different genus, and the herbaceous plant lettuce, to validate the model. The results may have certain limitations when applied to the estimation of leaf water content in other plant species. Future research should further collect leaf samples from other plant species to evaluate the feasibility of the model in estimating leaf water content across a variety of plants. The study collected peach tree leaves during two growth periods with the highest water content being 76.61%. Future research should collect data when leaf water content is higher, such as after rainfall or irrigation, and also collect data across other growth stages to build a more comprehensive dataset.

## 5 Conclusion

To achieve accurate estimation of leaf water content across different woody plant species and to supplement the validation across different growth forms, this study obtained hyperspectral data in the near-infrared region of peach tree leaves. estimation models for peach tree leaf water content were established using two methods: “vegetation indices” and “characteristic wavelengths.” The distribution of water content in peach tree leaves was visualized. The generalization ability of the optimal model was validated using the water content data of apple tree leaves (same family but different genus) and lettuce leaves (cross-growth form). The main conclusions are as follows:

1. Among the models established using the “vegetation index” method, the best-performing model was the linear regression model based on the vegetation index NISDI constructed in this study (R_P_^2^ = 0.9636, RMSEP=0.0356, using 3 wavelengths);
2. Among the models established using the “characteristic wavelength” method, the best-performing model was the CARS-RF model (R_P_^2^ = 0.9861, RMSEP=0.0219, using 3 wavelengths). Compared with the NISDI-based model, ΔR_P_^2^ = 0.0225 and ΔRMSEP=0.0131. However, the number of wavelengths used in modeling was significantly reduced from 12 to 3, a decrease of 75%. Therefore, the linear regression model based on NISDI was determined to be the optimal model;
3. When the water content and reflectance data of apple tree and lettuce leaves were input into the optimal model, the results showed that the R_P_^2^ and RMSEP for apple tree leaves were 0.9504 and 0.1226, respectively, while for lettuce, they were 0.5001 and 0.2694, respectively. When modeling was conducted separately for the leaf part of lettuce, the R_P_^2^ and RMSEP were 0.8211 and 0.1771, respectively. This suggests that the model possesses a certain degree of generalization capability within the same family of woody plants, but its performance experiences a decline when applied across different growth forms.

## Data Availability

The raw data supporting the conclusions of this article will be made available by the authors, without undue reservation.

## References

[B1] Araújo M. C. U. Saldanha T. C. B. Galvao R. K. H. Yoneyama T. Chame H. C. Visani V. (2001). The successive projections algorithm for variable selection in spectroscopic multicomponent analysis. Chemometrics Intelligent Lab. Syst. 57, 65–73. doi: 10.1016/s0169-7439(01)00119-8

[B2] Arya G. C. Sarkar S. Manasherova E. Aharoni A. Cohen H. (2021). The plant cuticle: an ancient guardian barrier set against long-standing rivals. Front. Plant Sci. 12. doi: 10.3389/fpls.2021.663165 PMC8267416 34249035

[B3] Bai S. H. Tootoonchy M. Kämper W. Tahmasbian I. Farrar M. B. Boldingh H.. (2024). Predicting carbohydrate concentrations in avocado and macadamia leaves using hyperspectral imaging with partial least squares regressions and artificial neural networks. Remote Sens. 16, 17. doi: 10.3390/rs16183389

[B4] Barnes R. Dhanoa M. S. Lister S. J. (1989). Standard normal variate transformation and de-trending of near-infrared diffuse reflectance spectra. Appl. Spectrosc. 43, 772–777. doi: 10.1366/0003702894202201

[B5] Breiman L. (2001). Random forests. Mach. Learn. 45, 5–32. doi: 10.1016/b978-0-12-824271-1.00018-4

[B6] Centner V. Massart D.-L. de Noord O. E. de Jong S. Vandeginste B. M. Sterna C. (1996). Elimination of uninformative variables for multivariate calibration. Analytical Chem. 68, 3851–3858. doi: 10.1021/ac960321m 21619260

[B7] Champagne C. M. Staenz K. Bannari A. McNairn H. Deguise J.-C. (2003). Validation of a hyperspectral curve-fitting model for the estimation of plant water content of agricultural canopies. Remote Sens. Environ. 87, 148–160. doi: 10.1016/s0034-4257(03)00137-8

[B8] Chu X. Yuan H. Lu W. (2004). Progress and application of spectral data pretreatment and wavelengthSelection methods in NIR analytical technique. Prog. Chem. 16, 528–542. doi: 10.3321/j.issn:1005-281X.2004.04.008

[B9] Dai Q. F. Liao C. L. Li Z. Song S. R. Xue X. Y. Xiong S. L. (2022). Hyperspectral visualization of citrus leaf moisture content based on CARS-CNN. Spectrosc. Spectral Anal. 42, 2848–2854. doi: 10.3964/j.issn.1000-0593(2022)09-2848-07

[B10] Dong C. W. An T. Yang M. Yang C. S. Liu Z. Y. Li Y.. (2022). Quantitative prediction and visual detection of the moisture content of withering leaves in black tea (Camellia sinensis) with hyperspectral image. Infrared Phys. Technol. 123, 10. doi: 10.1016/j.infrared.2022.104118

[B11] Dou S. Q. Zhang W. J. Deng Y. X. Zhang C. H. Mei Z. M. Yan J. C.. (2024). Comparison of citrus leaf water content estimations based on the continuous wavelet transform and fractional derivative methods. Horticulturae 10, 15. doi: 10.3390/horticulturae10020177

[B12] Ellsworth P. Z. V E. P. A. M. R. K K. N. & B. C. A. (2023). Leaf cell wall properties and stomatal density influence oxygen isotope enrichment of leaf water. Plant Cell Environ. 46, 2694–2710.doi:10.1111/PCE.14612. doi: 10.1111/pce.14612 37219338

[B13] Gao B.-C. (1995). Normalized difference water index for remote sensing of vegetation liquid water from space. Defense Security Sens. 2480, 225–236. doi: 10.1117/12.210877

[B14] Geladi P. Kowalski B. R. (1986). Partial least-squares regression: a tutorial. Analytica chimica Acta 185, 1–17. doi: 10.1016/0003-2670(86)80028-9

[B15] Geladi P. MacDougall D. Martens H. (1985). Linearization and scatter-correction for near-infrared reflectance spectra of meat. Appl. Spectrosc. 39, 491–500. doi: 10.1366/0003702854248656

[B16] Guo Y. Guo J. Shi Y. Li X. Huang H. Liu Y. (2022). Estimation of leaf moisture content in cantaloupe canopy based on siPLS-CARS and GA-ELM. Spectrosc. Spectral Anal. 42, 2565–2571. doi: 10.3964/j.issn.1000-0593(2022)08-2565-07

[B17] Horablaga A. Sibu A. Megyesi C. I. Gligor D. Bujanca G. S. Velciov A. B.. (2023). Estimation of the Controlled Release of Antioxidants from β-Cyclodextrin/Chamomile (Matricaria chamomilla L.) or Milk Thistle (Silybum marianum L.), Asteraceae, Hydrophilic Extract Complexes through the Fast and Cheap Spectrophotometric Technique. Plants-Basel 12, 24. doi: 10.3390/plants12122352 PMC10303029 37375976

[B18] Jobson J. Jobson J. (1991). Multiple linear regression. Appl. multivariate Data analysis: Regression Exp. design, 219–398. doi: 10.1038/nmeth.3665

[B19] Jones C. L. Weckler P. R. Maness N. O. Stone M. L. Jayasekara R. (2004). Estimating water stress in plants using hyperspectral sensing. 2004 ASAE Annual Meetin. Am. Soc. Agric. Biol. Engineers 1. doi: 10.13031/2013.17087

[B20] Junttila S. Holtta T. Saarinen N. Kankare V. Yrttimaa T. Hyyppa J.. (2022). Close-range hyperspectral spectroscopy reveals leaf water content dynamics. Remote Sens. Environ. 277, 13. doi: 10.1016/j.rse.2022.113071

[B21] Li H. Liang Y. Xu Q. Cao D. (2009). Key wavelengths screening using competitive adaptive reweighted sampling method for multivariate calibration. Analytica chimica Acta 648, 77–84. doi: 10.1016/j.aca.2009.06.046 19616692

[B22] Liu Y. Zhou X. Sun J. Li B. Ji J. Y. (2024). A method for non-destructive detection of moisture content in oilseed rape leaves using hyperspectral imaging technology. J. Nondestructive Eval. 43, 12. doi: 10.1007/s10921-024-01049-w

[B23] Lu Y. Y. Yang G. Shen Y. J. Yang H. Y. Xu K. C. (2022). Multifunctional flexible humidity sensor systems towards noncontact wearable electronics. Nano-Micro Lett. 14, 34. doi: 10.1007/s40820-022-00895-5 PMC9307709 35869398

[B24] Luo W. T. Gonzalez E. Zarei A. Calleja S. Rozzi B. Demieville J.. (2024). Leaf cuticular wax composition of a genetically diverse collection of lettuce (Lactuca sativa L.) cultivars evaluated under field conditions. Heliyon 10, 12. doi: 10.1016/j.heliyon.2024.e27226 PMC10923717 38463774

[B25] Lv S. Y. Wang J. H. Wang Z. D. Fang Y. Wang S. S. Wang F. Y.. (2024). Construction of hyperspectral reflectance and spectral index inversion model for the water content of Catalpa bungei leaves. Microchemical J. 197, 14. doi: 10.1016/j.microc.2023.109811

[B26] McCulloch W. S. Pitts W. (1943). A logical calculus of the ideas immanent in nervous activity. Bull. Math. biophysics 5, 115–133. doi: 10.1007/bf02478259 2185863

[B27] Norris K. H. Hart J. R. (1996). Direct spectrophotometric determination of moisture content of grain and seeds. J. Near Infrared Spectrosc. 4, 23–30. doi: 10.1255/jnirs.940

[B28] Peng Y. Q. Xiao Y. X. Fu Z. T. Dong Y. H. Li X. X. Yan H. J.. (2020). Water content detection of maize leaves based on multispectral images. Spectrosc. Spectral Anal. 40, 1257–1262. doi: 10.3964/j.issn.1000-0593(2020)04-1257-06

[B29] Qu F. F. Nie P. C. Lin L. Cai C. Y. He Y. (2018). Review of theoretical methods and research aspects for detecting leaf water content using terahertz spectroscopy and imaging. Int. J. Agric. Biol. Eng. 11, 27–34. doi: 10.25165/j.ijabe.20181105.3952

[B30] Reynoud N. Petit J. Bres C. Lahaye M. Rothan C. Marion D.. (2021). The complex architecture of plant cuticles and its relation to multiple biological functions. Front. Plant Sci. 12. doi: 10.3389/fpls.2021.782773 PMC8702516 34956280

[B31] Savitzky A. Golay M. J. (1964). Smoothing and differentiation of data by simplified least squares procedures. Analytical Chem. 36, 1627–1639. doi: 10.1021/ac60214a047 22324618

[B32] Shu M. Y. Dong Q. Z. Fei S. P. Yang X. H. Zhu J. Y. Meng L.. (2022). Improved estimation of canopy water status in maize using UAV-based digital and hyperspectral images. Comput. Electron. Agric. 197, 11. doi: 10.1016/j.compag.2022.106982

[B33] Sun H. Feng M. C. Xiao L. J. Yang W. D. Wang C. Jia X. Q.. (2019). Assessment of plant water status in winter wheat (Triticum aestivum L.) based on canopy spectral indices. PloS One 14, 15. doi: 10.1371/journal.pone.0216890 PMC6557497 31181067

[B34] Sun H. Ning L. Li W. Tao Z. Min-zan L. Jing-zhu W. (2018). Visualization of water content distribution in potato leaves based on hyperspectral image. 光谱学与光谱分析 39, 910–916. doi: 10.3964/j.issn.1000-0593(2019)03-0910-07

[B35] Wang Z. Q. Huang H. Wang H. Peñuelas J. Sardans J. Niinemets Ü.. (2022). Leaf water content contributes to global leaf trait relationships. Nat. Commun. 13, 9. doi: 10.1038/s41467-022-32784-1 36130948 PMC9492732

[B36] Wang Q. Li P. (2012). Identification of robust hyperspectral indices on forest leaf water content using PROSPECT simulated dataset and field reflectance measurements. Hydrological processes 26, 1–12. doi: 10.1002/hyp.8221

[B37] Wang L. J. Yang Y. Y. (2017). Purification and noise elimination of near infrared spectrum in rapid detection of milk components concentration by using principal component weight resetting. Acta Optica Sin. 37, 350–357. doi: 10.3788/AOS201737.1030003

[B38] Xu M. X. Wu S. H. Zhou S. L. Liao F. Q. Ma C. M. Zhu C. (2011). Hyperspectral reflectance models for retrieving heavy metal content: application in the archaeological soil. J. Infrared Millimeter Waves 30, 109–114. doi: 10.3724/SP.J.1010.2011.00109

[B39] Yang Y. C. Nan R. Mi T. X. Song Y. X. Shi F. H. Liu X. R.. (2023b). Rapid and nondestructive evaluation of wheat chlorophyll under drought stress using hyperspectral imaging. Int. J. Mol. Sci. 24, 15. doi: 10.3390/ijms24065825 PMC10056805 36982900

[B40] Yang B. C. Zhang H. N. Lu X. H. Wan H. L. Zhang Y. Zhang J.. (2023a). Inversion of leaf water content of cinnamomum camphora based on preferred spectral index and machine learning algorithm. Forests 14, 20. doi: 10.3390/f14122285

[B41] Yasir Q. M. Zhang Z. J. Tang J. K. Naveed M. Jahangir Z. (2023). Spectral indices for tracing leaf water status with hyperspectral reflectance data. J. Appl. Remote Sens. 17, 19. doi: 10.1117/1.Jrs.17.014523

[B42] Yuwei H. Yanli L. Liping Y. Guoshun Y. Kunyu L. Lei W. (2021). Hyperspectral response characteristics and correlation analysisof grape leaf tissue structure. J. Plant Nutr. Fertilizers 27, 1213–1221. doi: 10.11674/zwyf.20571

[B43] Zhang C. Li C. He M. Y. Cai Z. Y. Feng Z. P. Qi H. N.. (2023). Leaf water content determination of oilseed rape using near-infrared hyperspectral imaging with deep learning regression methods. Infrared Phys. Technol. 134, 8. doi: 10.1016/j.infrared.2023.104921

